# Gaining metabolic insight in older men undergoing androgen deprivation therapy for prostate cancer (the ADT & Metabolism Study): Protocol of a longitudinal, observational, cohort study

**DOI:** 10.1371/journal.pone.0281508

**Published:** 2023-02-10

**Authors:** Milena Braga-Basaria, Thomas G. Travison, Mary-Ellen Taplin, Alexander Lin, Alyssa B. Dufour, Daniel Habtemariam, Paul L. Nguyen, Adam S. Kibel, Praful Ravi, Richelle Bearup, Hannah Kackley, Hussein Kafel, Kieran Reid, Thomas Storer, Donald C. Simonson, Marie McDonnell, Shehzad Basaria

**Affiliations:** 1 Division of Endocrinology, Diabetes and Hypertension, Brigham and Women’s Hospital, Harvard Medical School, Boston, Massachusetts, United States of America; 2 Department of Medicine, Hinda and Arthur Marcus Institute for Aging Research, Hebrew SeniorLife, Beth Israel Deaconess Medical Center, Harvard Medical School, Boston, Massachusetts, United States of America; 3 Lank Center for Genitourinary Oncology and Dana-Farber Cancer Institute, Harvard Medical School, Boston, Massachusetts, United States of America; 4 Department of Radiology, Center for Clinical Spectroscopy, Brigham and Women’s Hospital and Dana Farber Cancer Institute, Harvard Medical School, Boston, Massachusetts, United States of America; 5 Department of Radiation Oncology, Brigham and Women’s Hospital and Dana Farber Cancer Institute, Harvard Medical School, Boston, Massachusetts, United States of America; 6 Division of Urology, Department of Surgery, Brigham and Women’s Hospital, Harvard Medical School, Boston, Massachusetts, United States of America; PhD, PLOS, UNITED KINGDOM

## Abstract

Androgen deprivation therapy (ADT), a cornerstone of treatment for patients with locally advanced and metastatic prostate cancer, is associated with many adverse effects, including osteoporosis, sexual dysfunction, fatigue, and vasomotor symptoms. It is also associated with loss of muscle mass and increased adiposity. This change in body composition is likely the inciting event in the development of insulin resistance, an independent risk factor for diabetes mellitus and cardiovascular disease. Although the occurrence of insulin resistance during ADT has been reported, it remains unclear whether this insulin resistance is primarily hepatic or muscular. Similarly, the mechanisms that lead to insulin resistance also remain unknown. The ADT & Metabolism Study was designed to address these knowledge gaps, as the elucidation of the predominant site of insulin resistance will allow prevention strategies and the use of targeted, tissue-specific insulin-sensitizing agents in patients undergoing ADT. This prospective, mechanistic, single-center, 24-week, observational cohort study will enroll treatment-naïve adult men with prostate cancer about to undergo surgical or medical ADT for at least 24 weeks (ADT group; n = 50) and a control group of men who had undergone radical prostatectomy and are in remission (non-ADT group, n = 25). The primary outcome is to determine the site of insulin resistance (skeletal muscle or liver) using frequent sampling oral glucose tolerance test at baseline and 12 and 24 weeks after commencement of ADT (ADT group) or after enrollment in the study (non-ADT group). Secondary outcomes will assess changes in hepatic and intramyocellular fat (using magnetic resonance spectroscopy), inflammatory markers, adipokines, free fatty acids, and changes in body composition (assessed using dual-energy x-ray absorptiometry) and their correlation with the development of insulin resistance. Exploratory outcomes will include changes in muscle performance, physical function, physical activity, vitality, and sexual drive.

## Introduction

Prostate cancer is the most common non-cutaneous cancer in men in the United States [1]. At initial diagnosis, the majority of men have early-stage disease and are generally managed with surveillance or local approaches including prostatectomy and radiation; however, for patients with locally advanced, recurrent, or metastatic disease, androgen deprivation therapy (ADT) remains the cornerstone of treatment. The rationale for using ADT in these patients stems from the fact that prostate cancer is dependent on androgens for growth [2]. In addition to the indications mentioned above, ADT is also being used in some patients with early-stage prostate cancer and in those with biochemical recurrence (rising prostate-specific antigen [PSA] levels following initial remission after prostatectomy). Either surgical (orchiectomy) or medical (therapy with gonadotropin-releasing hormone [GnRH] agonists or antagonists), ADT decreases serum testosterone levels into the castrate range (<20 ng/dL [0.6934 nmol/L]). The profound androgen deficiency that occurs as a consequence of ADT not only leads to sexual dysfunction, hot flashes and osteoporosis [3], but it also results in a significant increase in fat mass (both subcutaneous and "metabolically active" visceral fat) and loss of muscle mass [4, 5]. These unfavorable changes in body composition are the seminal events that likely lead to the development of insulin resistance, an independent risk factor for atherosclerosis and cardiovascular disease (CVD) [6–9] and a precursor to diabetes and metabolic syndrome [10–12].

Previous studies have reported that ADT is associated with insulin resistance, diabetes, and metabolic syndrome [13–17] and that these metabolic perturbations display an accelerated course in androgen-deprived men as insulin resistance develops as early as 6–8 weeks after initiation of ADT [18–20]. These metabolic changes can predispose patients to CVD, a prominent cause of mortality in men undergoing ADT [21, 22]. Indeed, men on ADT, compared with those not receiving ADT, have higher risk of incident coronary artery disease, myocardial infarction, peripheral vascular disease, and cardiovascular mortality. [23, 24]. Additionally, men with known CVD are at the highest risk of further complications.

Systemic insulin resistance is a sum of insulin resistance occurring in different organs, predominantly the liver and the skeletal muscle [25–29]. Although the occurrence of insulin resistance in men on ADT has been reported previously, the predominant site of insulin resistance (muscle or liver) and the mechanisms behind its development (role of parenchymal fat infiltration and inflammatory cytokines) remain unclear. Elucidation of these mechanisms will provide opportunity for prevention and possible use of novel tissue-specific insulin-sensitizing agents that prevent development of insulin resistance in these patients.

The current study is designed to address these knowledge gaps and overcome the limitations of prior studies [30]. The primary aim of this study is to determine the predominant site of insulin resistance in men with prostate cancer undergoing ADT by performing a state-of-the-art, frequent sampling, oral glucose tolerance test (OGTT) that will determine whether insulin resistance that develops as a consequence of profound hypogonadism induced by ADT is predominately at the level of the liver or skeletal muscles. Secondary aims focus on determining the mechanisms behind this insulin resistance by assessing changes in hepatic and intramyocellular fat (using magnetic resonance spectroscopy [MRS]), changes in body composition (on dual x-ray absorptiometry [DEXA] scan) and measurement of circulating inflammatory cytokines. Exploratory aims will include assessment of muscle performance, physical function, energy (vitality), and sexual function.

## Materials and methods

### Study design and setting

This study protocol was approved by the Institutional Review Board at Dana-Farber Cancer Institute. The study is funded by the National Cancer Institute (5R01CA226211).

This is a prospective, single-center, 24-week, mechanistic observational cohort study enrolling hormone-naïve (no prior ADT) adult men with prostate cancer who are about to undergo surgical or medical ADT for at least 24 weeks (ADT group). The control group will comprise men with prior history of prostatectomy for localized prostate cancer who are in remission, are hormone-naïve, and have no planned ADT (non-ADT group). Participants will be recruited from the Dana Farber Cancer Institute and Brigham and Women’s Hospital in Boston, Massachusetts, USA.

### Outcomes

**Fig 1:**
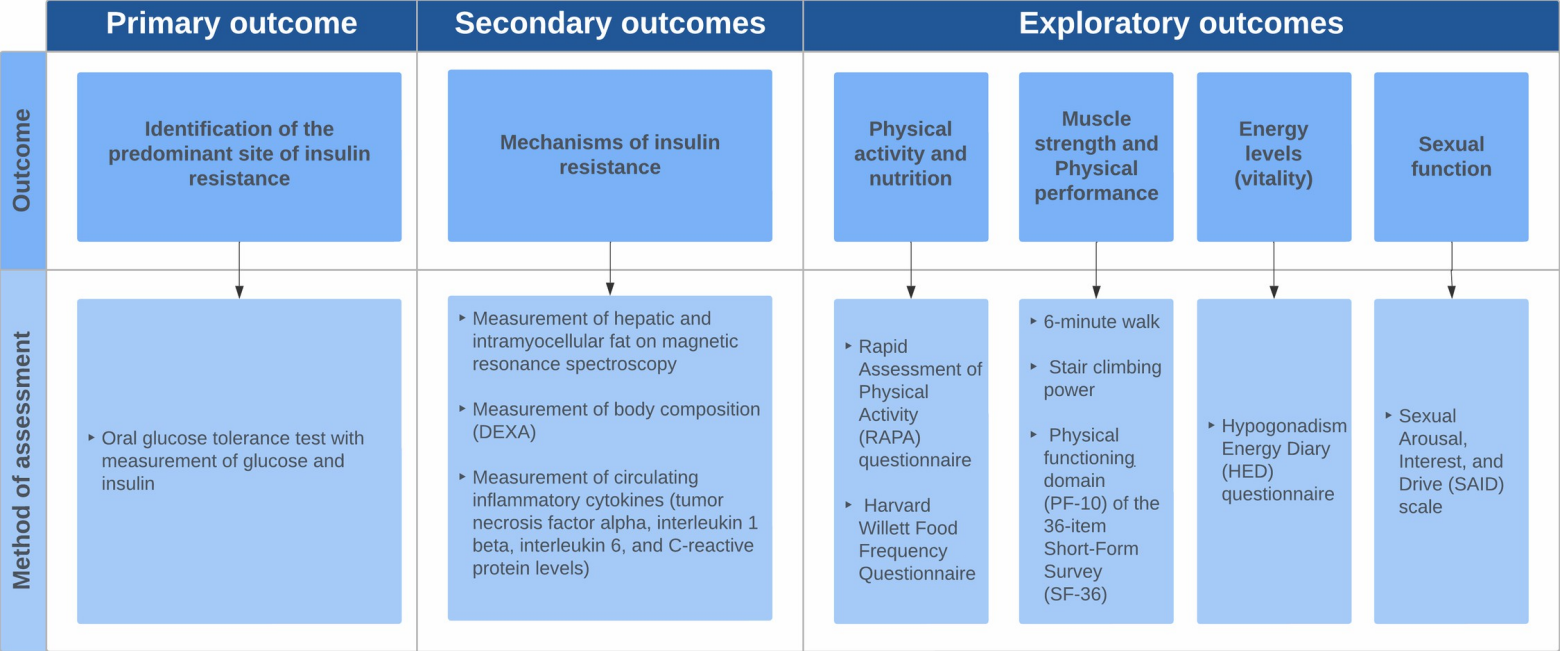
Primary, secondary, and exploratory outcomes of the study.

Fig 1 shows the primary, secondary, and exploratory outcomes of the study.

### Eligibility criteria

**Fig 2:**
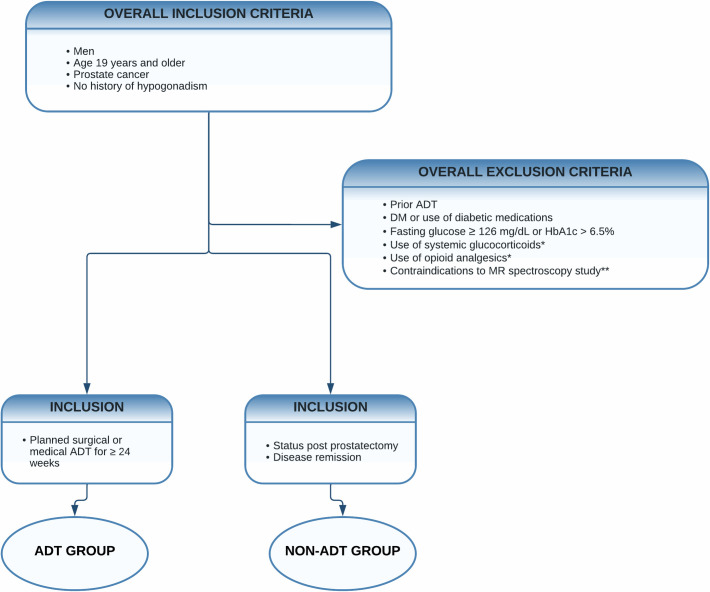
Eligibility criteria for participants. Abbreviations: ADT, androgen deprivation therapy; DM, diabetes mellitus; HbA1c, glycated hemoglobin; MR spectroscopy, magnetic resonance spectroscopy. *Within 3 months from enrollment. ** Participants who have contraindications to MRS but are otherwise eligible will be enrolled.

Fig 2 summarizes the eligibility criteria for each group.

### Schedule of study procedures

**Table 1** shows a schedule of enrollment and assessments.

**Table 1:** Schedule of enrollment and assessments.

Procedures	Screening	Baseline	12 weeks	24 weeks
Consent/Eligibility	X			
Medical history	X			
Vital signs, electrocardiogram, anthropometry (body mass index, waist/hip ratio)	X	X	X	X
Screening labs (fasting blood glucose, hemoglobin A1c, total testosterone)	X			
Physical exam	X			
Oral glucose tolerance test		X	X	X
Magnetic resonance spectroscopy (liver and calf muscles)		X		X
Body composition (dual-energy x-ray absorptiometry)		X	X	X
Total and free testosterone, sex hormone binding globulin, luteinizing hormone.		X	X	X
Inflammatory cytokines (C-reactive protein, tumor necrosis factor-α, interleukin-1β, interleukin-6), adipokines (leptin, adiponectin, resistin), free fatty acids		X	X	X
Complete blood count, CMP, PSA		X	X	X
Fasting lipid profile, hemoglobin A1c		X	X	X
Questionnaires (RAPA, HWFF, HED, SAID, PF-10)		X	X	X
Muscle strength, and physical function assessments (leg press strength and power/6-min walk test /loaded stair climb power)		X	X	X

Abbreviations: CMP, comprehensive metabolic panel; PSA, prostate-specific antigen; RAPA, Rapid Assessment of Physical Activity Questionnaire; HWFF, Harvard Willett Food Frequency Questionnaire; HED, Hypogonadism Energy Diary; SAID, Sexual Arousal, Interest, and Drive scale; PF-10 (physical function domain of the 36-Item Short-Form Survey).

### Screening visit

Participants who are eligible on phone screening will be invited for an in-person screening visit. At the screening visit, participants will be consented and assessed for eligibility. At this visit, we will obtain medical history, review current medications, check vital signs, evaluate anthropometric measurements, perform a physical exam, and obtain an electrocardiogram (ECG). Fasting morning labs will be drawn to check glucose, hemoglobin A1c (HbA1c), and testosterone. Participants who conform to all eligibility criteria will be enrolled.

### Baseline, 12 weeks, and 24 weeks visits

The study procedures (Table 1) will be performed at the baseline, 12-weeks, and 24-week visits, except for MRS, which will be performed at baseline and 24-week visits. For the ADT group, the 12- and 24-week visits will be timed from the date of commencement of ADT. For the non-ADT group, these visits will be timed from the baseline visit. If a participant’s 2-hour glucose value during OGTT at the baseline visit is ≥200 mg/dL (11.1 mmol/L), he will be excluded, as this meets the criterion for prevalent diabetes [31].

#### Oral glucose tolerance test

All participants will undergo frequent sample OGTT according to the technique developed by Matsuda & DeFronzo [32] and Abdul-Ghani et al. (33). This state-of-the-art OGTT was developed to quantify "tissue-specific" insulin resistance that is comparable, and less burdensome, to the hyperinsulinemic euglycemic clamp technique. The procedure entails collection of samples for fasting glucose and insulin at -30, -15, and 0 minutes followed by administration of 75 grams of glucose and collection of samples at 30, 60, 90, and 120 minutes. Plasma glucose will be quantified using a Beckman Glucose Analyzer and serum insulin will be measured using high-sensitivity sandwich enzyme-linked immunosorbent assay (ELISA).

#### Hepatic and muscle insulin resistance

The glucose load administered in the OGTT is followed by a rise in plasma glucose concentrations, which, in turn, stimulates secretion of insulin by the pancreatic beta cells. In individuals with normal insulin sensitivity, insulin secretion suppresses hepatic glucose output in the first 30 minutes of the OGTT. This increase in insulin triggers glucose disposal into peripheral tissues, the predominant tissue being the skeletal muscle. Thus, the decline in glucose levels from peak value primarily reflects glucose uptake by the skeletal muscle.

**Fig 3:**
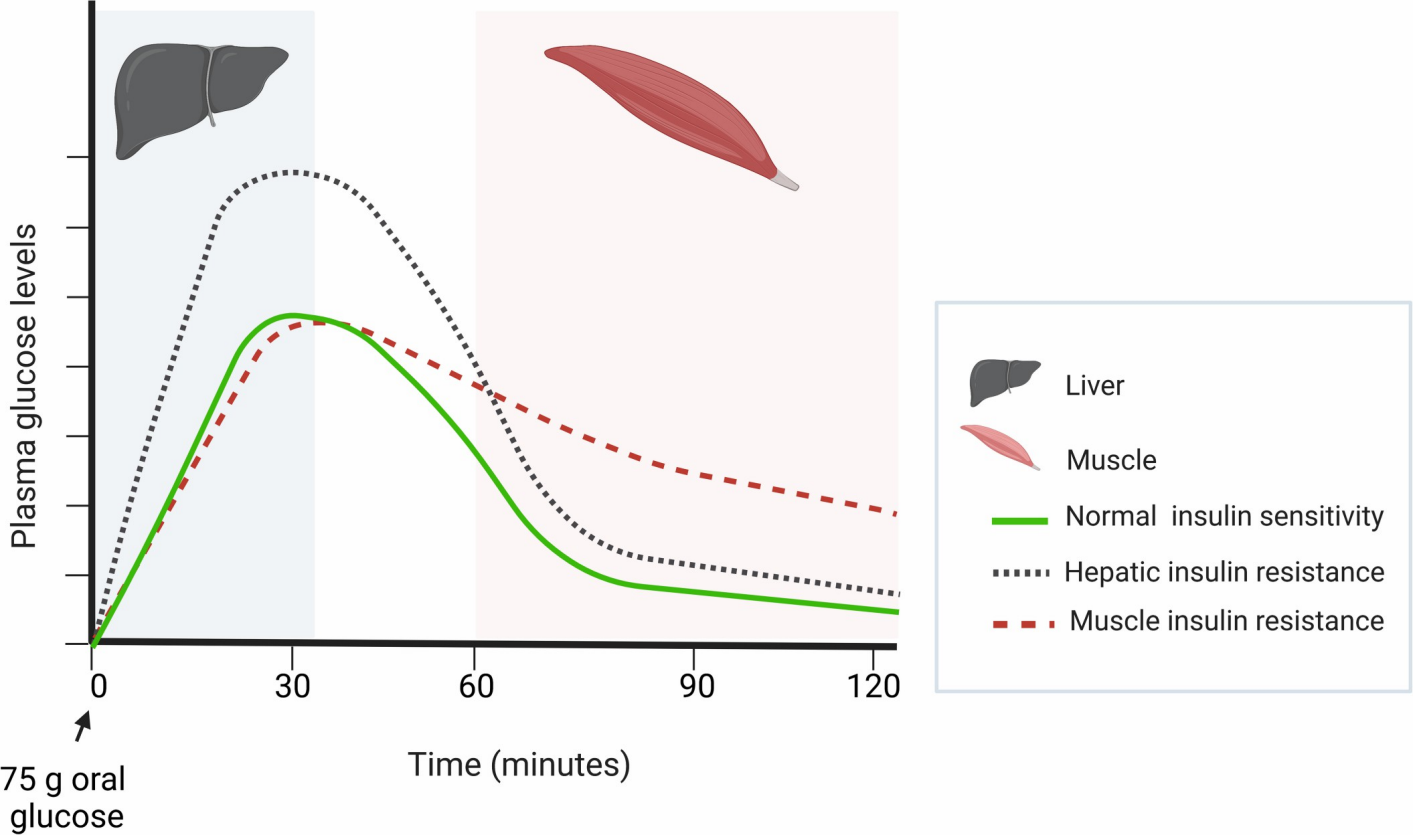
Anticipated patterns of changes in plasma glucose concentrations according to the primary site of insulin resistance. * *Based on information from Reference #33.

In individuals with hepatic insulin resistance, insulin is unable to suppress hepatic glucose output efficiently. In such subjects, a higher peak of glucose is observed in the first 30 minutes of the OGTT (*i*.*e*., a sum of endogenous and exogenous glucose), and the magnitude of the rise in plasma glucose and insulin concentrations during this period is proportional to the degree of the hepatic insulin resistance. If skeletal muscle insulin sensitivity in these individuals is normal, glucose is normally disposed into peripheral tissues, and the glucose peak is followed by a steady decline. In contrast, in individuals with predominantly skeletal muscle insulin resistance, insulin secretion efficiently suppresses hepatic glucose output in the first 30 minutes of the OGTT, but peripheral disposal of glucose is impaired. Thus, these subjects display a slower decline in glucose levels from peak value (Fig 3).

In the present study, we will quantify hepatic insulin resistance for each subject using the product of the area-under-the-curve (AUC) for insulin and glucose concentrations during the first 30 minutes (0–30) of the OGTT, according to the equation proposed by Abdul-Ghani et al. [33]:

$$Glucose_{0-30}(AUC)\times Insulin_{0-30}(AUC)$$

Skeletal muscle insulin resistance will be assessed based on the rate of decline of plasma glucose with time, calculated as the slope of the least square fit to the decline in plasma glucose concentration from peak to nadir (dG/dt) divided by the mean plasma insulin concentration (I) [33]:

(dG/dt)÷meanplasmainculinconcentration

For each of these calculations, the change from baseline to 12 and 24 weeks will be computed at the subject level, and analyses will be performed on the resulting change scores.

#### Magnetic resonance spectroscopy

As a noninvasive imaging method for measuring concentrations of intracellular metabolites, MRS offers insight into the fat content in various tissues [34]. In the ADT & Metabolism Study, enrolled participants will undergo MRS for quantification of hepatic and skeletal muscle fat. The MRS will be performed using a 3-Tesla magnetic resonance imaging scanner (Skyra VE11C; Siemens AG, Erlangen, Germany). Hepatic and skeletal muscle MRI and MRS will be acquired with the following steps:

Hepatic MRS
Subjects will be imaged head first using a phased array torso coil centered at the liver. Plane breath held localizers will be followed by coronal and transverse T2 HAlf fourier Single-shot Turbo spin-Echo (HASTE) for localization of the MRS voxels. Two voxels will be acquired in the posterior and anterior portions of the liver avoiding vasculature and non-hepatic structures. A screenshot will be taken at the baseline scan, which will then be referenced in the follow-up scans to ensure reproducibility of the voxel location between the baseline and follow-up scan. MRS will be acquired using breath-held stimulated echo acquisition mode (STEAM) with an echo time (TE) of 20 ms, repetition time (TR) of 2 seconds, mixing time of 10 ms, 8 averages (16 seconds) with a voxel size of 2x2x2 cm^3^. Automated shimming will be used but if linewidths are greater than 30 hz, manual shimming will be conducted to minimize linewidths. Spectroscopy data will be exported offline in raw data mode (twix) for post-processing. Two areas of interest will be selected to quantitate hepatic fat. Intensities of the peaks resonating from the protons of hepatic water (4.7 ppm) and protons of methylene groups (-CH2-) in the fatty acid chains (1.3 ppm) will be determined (corrected for T1 and T2 relaxation), and liver fat content will be expressed as a ratio of the signal of the methylene group to the total signal of methylene plus water using LC model. Hepatic lipid content will be determined from the relative percentage of the spectral peak at 1.3 ppm (representing the sum of triglyceride acyl chains) over the spectral peak of protons from hepatic water at 4.7 ppm. To convert the relative quantity of hepatic fat and water into absolute concentrations, the method described by Szczepaniak et al. will be used, which has been widely accepted as the most accurate assessment of liver fat content [35].
Skeletal Muscle MRS
Muscle fat spectroscopy will be acquired in the same scanner following the hepatic MRS. The patient will lie supine, feet first, and the lower limb will be placed in a 15-channel transmit-receive knee coil. Special care will be taken to ensure that the subject’s leg is parallel with the bore for optimal separation of the intramyocellular lipid content (IMCL) and extramyocellular lipid content (EMCL) resonances. Three plane localizers will be acquired, followed by axial T1-weighted MRI with and without fat suppression. Proton MRS (1H-MRS) single voxel spectra using Point-RESolved Spectroscopy (PRESS) with a TR/TE = 2000/30 ms, 128 averages (4 min, 16 sec) will be acquired from the gastrocnemius and tibialis anterior muscles (1.5 x 1.5 x 2 cm^3^) for IMCL/EMCL analysis. Likewise, a screenshot will be taken for reference and to reproduce the same location. Scans will be processed using an automated time-domain-based spectral fitting software (LCModel v6.3, Provencher) which will output concentrations of IMCL, EMCL, and creatine.

#### Measurement of sex steroids, inflammatory cytokines, and adipokines

Inflammatory cytokines and adipokines will be measured in serum using high-sensitivity sandwich ELISA. Total testosterone will be measured using liquid chromatography-tandem mass spectrometry (sensitivity of 2 ng/dL [0.0693 nmol/L]), and free testosterone will be measured with equilibrium dialysis. Sex hormone binding globulin will be measured by solid phase immunofluorometric assay. Except for sex steroids (which will be measured in real time), inflammatory cytokines and adipokines will be measured on frozen plasma and sera will be banked in a -80°C freezer.

#### Dual-energy X-ray absorptiometry

Participants will undergo assessment of body composition using DEXA scans (Hologic QDR 4500A; Hologic, Inc., Bedford, Massachusetts, USA). Both lean body mass and fat mass (subcutaneous and visceral adipose tissue) will be measured. The scanner will be regularly calibrated using a soft-tissue phantom.

#### Physical activity and nutrition assessment

The participants’ physical activity will be assessed using the validated Rapid Assessment of Physical Activity (RAPA) questionnaire, a one-page tool that has been used in several clinical trials [36, 37]. Nutrition intake will be assessed using the Harvard Willett Food Frequency Questionnaire, which has been extensively validated in many populations [38–40]. These assessments will be performed in both cohorts at baseline, 12, and 24 weeks.

#### Muscle performance and physical function

Maximal voluntary leg extensor muscle strength will be assessed using the one-repetition maximum test during a seated leg-press exercise (Keiser Corporation, Fresno, CA) [41]. Leg extensor muscle power will be assessed using the same equipment and positioning used for the 1-RM assessment, as previously described [42]. Physical function will be assessed by (1) measurement of distance walked during the 6-minute walk test [43], (2) loaded stair climbing power test [44], and (3) self-reported physical function assessed by the physical functioning domain of the 36-item Short-Form Survey (PF-10).

#### Energy and sexual function

The participant’s energy (vitality) and sexual desire will be assessed using the validated Hypogonadism Energy Diary (HED) questionnaire and the Sexual Arousal, Interest, and Drive (SAID) scale [45], respectively.

### Measurement of effect

The primary outcome is to determine the predominant site of insulin resistance (liver or skeletal muscle) in enrolled participants using the state-of-the-art OGTT.

Secondary outcomes include assessment of (1) intramyocellular and hepatic fat using MRS, (2) inflammatory cytokines, and (3) body composition.

Additional analyses will explore changes between groups in muscle strength and physical performance (leg press strength, 6-minute walk, loaded stair climb power), physical activity (PF-10 and RAPA), and sexual function/vitality (SAID, HED).

### Statistical analysis

The primary analyses will focus on differences between the ADT and non-ADT groups, while secondary analyses will consider ADT exposure and other factors that are only specific to ADT. To ensure adequate statistical power for the ADT-specific analyses, we will enroll twice as many subjects in the ADT group as in the non-ADT group.

#### Quantification of primary outcome (Specific Aim 1)

Differentiation between insulin resistance occurring at the liver or skeletal muscle between baseline and 12- and 24 weeks will be obtained from the two-hour OGTT.

Among subjects exhibiting insulin resistance at the liver or skeletal muscle, circulating glucose (G) concentrations are expected to follow roughly the pattern displayed in Fig 3. For participants manifesting resistance predominantly at the liver, glucose concentrations are expected to peak at roughly 30 minutes and then decline steadily. Meanwhile, for participants expressing resistance predominantly at the muscle, concentrations are expected to decline less rapidly. Hepatic insulin resistance is quantified according to lack of suppression of hepatic glucose output in the initial period following start of OGTT. Therefore, following Abdul-Ghani et al. [33], we will quantify hepatic resistance using the product of area-under-the-curve (AUC) for insulin and glucose concentrations during the first 30 minutes of OGTT (glucose_0–30[AUC]_ X insulin_0–30[AUC]_) for each subject. Level of skeletal muscle insulin resistance is in rough (negative) proportion to the rate of decline of plasma glucose as time (t) progresses, dG/dt, during the period between glucose peak until reaching its nadir. We will, therefore, quantify skeletal muscle insulin resistance using estimated dG/dt divided by the mean plasma insulin concentration (I). For each of these, change from baseline to 12 and 24 weeks will be computed at the subject level, and analyses will be performed on the resulting change scores.

#### Quantification of secondary outcomes (Specific Aim 2)

Skeletal muscle and hepatic fat concentrations will be assessed from MRS (baseline and 24 weeks). Concentrations of inflammatory cytokines and adipokines will be determined using the methodology described above. Body composition will be assessed using DEXA scan (baseline, 12, and 24 weeks).

#### Overall analytic approach

Prior to formal analysis, variable distributions will be summarized by cohort (ADT and non-ADT groups). Means, standard deviations, ranges, medians, quartiles, and skewness will be determined. Graphical methods will be utilized to assess overall distributional properties. Scatterplot smoothing and exploratory assessments via generalized additive models will be employed to assess the functional form of associations between covariates and outcomes. All point estimates will be accompanied by 95% confidence intervals. Hypothesis tests will be evaluated at the 0.05 level. As hypotheses are prespecified, no adjustment will be made for multiple comparisons.

#### Aim-specific analytic plan

*Specific Aim 1*. Specific Aim 1 is concerned with determining whether the predominant site of insulin resistance is at the level of the liver and at the skeletal muscle using, respectively, glucose_0–30[AUC]_ X insulin_0–30[AUC]_) (denoted AUC hereafter) and dG/dt / I, as described above. These will be modeled separately. Each subject’s change in AUC, dG/dt and I will be computed, and change at 12 and 24 weeks for each obtained. Primary analyses will estimate change at 12 and 24 weeks simultaneously using a mixed-effects regression analysis with control for cohort membership [46, 47]. The differential increase in AUC with time accompanying ADT as compared to controls will be estimated using a treatment contrast and robust (employing sandwich variance estimators) 95% confidence interval, and quantification of statistical significance obtained using a likelihood ratio test. Analysis of dG/dt / I will proceed in like fashion.

Assuming stable cross-sectional standard deviation (SD) with time, the SD of change in outcomes is given by √2(1 − ρρ), where ρρ is the Pearson correlation between repeated outcome measures, which we anticipate will be high. Under the relatively conservative assumption that ρρ = 0.5, an evaluable sample size of 40 participants in the ADT group and 20 in the non-ADT group will provide 90% power to detect a mean between-group difference in AUC change and dG/dt / I change of 9.03 units and 1.35 units, respectively, and 80% power to detect differences of 7.8 units and 1.17 units, respectively, at either follow-up time. Under these assumptions, a total of 40 evaluable subjects would in turn provide adequate precision to estimate mean change in AUC and dG/dt / I to within 3.2 units and 0.47 units, respectively, using 95% confidence intervals. Based on an assumption of cumulative attrition and missingness of 20%, the trial will therefore enroll 50 participants in the ADT group and 25 in the non-ADT group, or N = 75 participants overall. The proposed sample size is intended to facilitate comparisons of the ADT to non-ADT groups in Specific Aims 1 and 2, as well as providing the necessary precision to estimate change in outcomes in analyses of the ADT group alone in Specific Aim 2.

*Specific Aim 2*. *Specific Aim 2 is concerned with assessment of fat deposition and inflammatory markers*.

*Analyses of Hepatic and Intramyocellular Fat*. These outcome measures will be obtained by MRS at baseline and 24 weeks. Change scores on each will be computed for each participant. In primary analyses, the mean within-subject change in fat measurements will be assessed, and between-group differences in change assessed using a point estimate and associated 95% confidence interval. In secondary analyses, the association between change and subject-level covariates including age, BMI, sex steroid levels, and lipids will be generated using a generalized additive model, which estimates trends and associated confidence regions using penalized likelihood. Where associations are sufficiently linear, multiple linear regression may be employed to generate parameters that are easily interpreted.

*Analyses of Inflammatory Cytokines*. As noted above, the proposed sample size of 50 participants in the ADT group (twice the size of the non-ADT group) is intended to facilitate evaluation of within-subject changes among participants exposed to ADT. Given projected attrition and missingness of 20%, we anticipate at least 40 evaluable change scores for any outcome and time point. In primary analyses, unadjusted changes in inflammatory cytokines will be estimated using a mixed-effects model as described above, with between-group differences estimated using a treatment contrast and robust confidence interval. Secondary analyses will estimate within-subject changes in each cohort and as a function of baseline age, BMI, and other covariates using generalized additive mixed models.

#### Additional analyses

Analyses paralleling those described above will be employed to assess differences in change in measures of physical function and muscle performance. Analyses of 6-minute walk distance and average speed will assess between-group differences using mixed-effects regression models and propensity scoring as described above. We will perform similar analyses to assess between group differences in leg press strength and power. This study will also assess loaded stair climbing power.

#### Sensitivity analysis and missing data

We anticipate that outcomes will exhibit reasonable conformity with assumptions, but will assess distributional properties, outlying observations, etc. as described above. In the case of outlying values, models will be re-estimated excluding these observations to ensure that qualitative conclusions are stable and robust. Where conformity with other modeling assumptions is threatened, robust alternatives such as analyses of rank-transformed change in outcome measures will be considered. We will model the missing data process as a repeated-measures binary outcome using the modified Poisson regression model [48].

#### Statistical computing

Analyses will be performed using SAS version 9.4 (SAS Institute, Cary, NC) or R version 3.1 or later (R Foundation for Statistical Computing, Vienna). Simultaneous modeling of missing data and repeated-measures outcomes will be performed using MPlus version 7 or later (Muthén and Muthén, Los Angeles, CA).

#### Ethical considerations and data sharing

Ethical approval has been obtained from the Institutional Review Board of Dana Farber Cancer Institute (Protocol # 18–442). Patients meeting the research criteria will be enrolled in the study after signing an informed consent. The study will be conducted in accordance with the principles of the Declaration of Helsinki and Good Clinical Practice. Following completion of the project, we will generate a data set suitable for data sharing purposes, which will be deidentified and stripped of protected health information according to the Health Insurance Portability and Accountability Act of 1996 (HIPAA) and Safe Harbor principles. The findings of the study will be published in peer-reviewed academic journals.

#### Data management plan

The study will utilize a secure electronic data capture (EDC) system. The database will be hosted on secure, password-protected servers.

The database will be fully HIPAA and 21 CFR part 11 compliant, providing full security and audit capabilities consistent with federal requirements and guidelines.

#### Status and timeline of the study or current status

Recruitment of participants commenced in February 2019, and data collection started in March 2019. The enrollment is now complete, and data collection is expected to be completed by April 2023. Data analysis will commence soon thereafter, and manuscripts will be published in 2023 and 2024.

## Discussion

The ADT & Metabolism Study is the first study designed to elucidate the predominant site of insulin resistance in men with prostate cancer undergoing ADT. Although the development of ADT-induced insulin resistance has been reported previously, the predominant site of insulin resistance (muscle or liver) remains unclear. What also remains unclear are the mechanisms that lead to ADT-induced insulin resistance. Data from population studies have shown that men with insulin resistance have higher serum concentrations of inflammatory cytokines and fat infiltration of parenchymal organs, such as the skeletal muscles and the liver [49–56]. Indeed, studies using Medicare database have shown that men undergoing ADT are at a higher risk of being diagnosed with nonalcoholic fatty liver disease compared with men who do not receive ADT [57]. However, it remains to be determined if deposition of fat in these organs contributes to the development of this insulin resistance. Unraveling the predominant site of insulin resistance will allow the use of novel, targeted, tissue-specific insulin-sensitizing agents in patients undergoing ADT. Similarly, the roles of parenchymal fat deposition and inflammatory cytokines in the pathogenesis of insulin resistance in this population deserves exploration, as newer drugs that antagonize action of these cytokines have been shown to improve insulin resistance in other populations [58–60]. As men undergoing ADT are old and frail [61, 62] with multiple comorbidities, the option of physical exercise is limited, and there is a great need for targeted treatment with novel insulin sensitizers. The findings of this study will lay the groundwork for prospective intervention trials with "tissue-specific" insulin-sensitizing agents, anti-inflammatory agents, and selective androgen receptor modulators (SARMs) [63] in preventing the development of insulin resistance during ADT, which in turn will lessen the burden of CVD in this population.

## Supporting information

S1 File (PDF)
