# Supplementary material for: Gaining metabolic insight in older men undergoing androgen deprivation therapy for prostate cancer (the ADT & Metabolism Study): Protocol of a longitudinal, observational, cohort study
Source: PLoS One. 2023 Feb 10;18(2):e0281508. doi: 10.1371/journal.pone.0281508 (PMC9916640; doi:10.1371/journal.pone.0281508)
Supplement: S1 File — (PDF) [file pone.0281508.s001.pdf]

DF/HCC BIOMEDICAL PROTOCOL

Version: December 11, 2019

NCI Protocol #: CA226211

DF/HCC Protocol #: 18-442

DF/HCC Biomedical Protocol: 12/11/2019

TITLE: Gaining Metabolic Insight in Older Men undergoing Androgen Deprivation Therapy for Prostate Cancer

Protocol Version Date: 12/11/2019 Version 4.0

Institution: Brigham and Women's Hospital

***Principal Investigator (PI):** Shehzad Basaria, MD

Brigham and Women's Hospital

Other Investigators:

Mary-Ellen Taplin, MD
Dana-Farber Cancer Institute

Paul Nguyen, MD
Dana-Farber Cancer Institute

Adam Kibel, MD
Brigham and Women's Hospital

Marie McDonnell, MD
Brigham and Women's Hospital

Alexander Lin, PhD
Brigham and Women's Hospital

Thomas G. Travison, PhD
Hebrew Senior Life

Thomas Storer, PhD
Brigham and Women's Hospital

Grace Huang, MD
Brigham and Women's Hospital

Shalender Bhasin, MD Brigham and Women's Hospital

Anna Goldman, MD
Brigham and Women's Hospital

Statistician:

Thomas G. Travison, PhD
Hebrew Senior Life
tgt@hsl.harvard.edu

Study Coordinators:

Richelle Bearup, MPH
Brigham and Women's Hospital
617-278-0722
FAX: 617-525-9148
rbearup@bwh.harvard.edu

Makaila Decker
Brigham and Women's Hospital
617-525-9153
FAX: 617-525-9148
mdecker1@bwh.harvard.edu

Michelle Blouin
Brigham and Women's Hospital
617-525-9095
FAX: 617-525-9148
mblouin@bwh.harvard.edu

Lauren Kelly
Brigham and Women's Hospital
617-525-9056
FAX: 617-525-9148
lekelly@bwh.harvard.edu

Julia Ravelson
Brigham and Women's Hospital
617-525-8407
FAX: 617-525-9148
jravelson@bwh.harvard.edu

Catherine Ghattas, MPH
Brigham and Women's Hospital
617-525-9198
FAX: 617-525-9148
cghattas@bwh.harvard.edu

Research Nurse:

Haley J. Schram, N.P.
Brigham and Women's Hospital
617-525-9196
FAX: 617-525-9148
hschram@ bwh.harvard.edu

Project Manager:

Brooke Ferguson Brawley, MPA
Brigham and Women's Hospital
617-525-9195
FAX: 617-525-9148
bbrawley@bwh.harvard.edu

Research Lab and Clinic Space Manager:

Yusnie Memish Beleva
Brigham and Women's Hospital
617-525-9152
FAX: 617-525-9148
ymemishbeleva@bwh.harvard.edu

Jenny Vo
Brigham and Women's Hospital
617-278-0564
FAX: 617-525-9148
Jvo2@bwh.harvard.edu

Jane Liva
Brigham and Women's Hospital
617-525-9150
FAX: 617-525-9148
jliva@bwh.harvard.edu

Table of Contents

Rationale and Objectives 6
Introduction 6
Study Design 6
Primary Objective 6
Secondary Objectives 7
Exploratory Objectives 7
Background 7
Factors Predisposing Men undergoing ADT to develop Metabolic Changes 8
Changes in Body Composition 8
Inflammation 8
Deposition of Hepatic and Intramyocellular Fat 9
CVD and Mortality 9
Rationale 9
Participant Selection 10
Eligibility Criteria 10
Inclusion Criteria for ADT-group 10
Inclusion Criteria for Non-ADT Patients 10
Common Exclusion Criteria for both Cohorts 10
Rationale for the Study Duration 11
Schedule of Study Procedures 11
Screening, Recruitment and Enrollment 11
Recruitment of ADT-group from Oncology Clinics at the Dana-Farber Cancer Institute (DFCI) 11
Recruitment of Non-ADT group from Urology Clinics at Brigham and Women's Hospital... 12
Inclusion of Minorities 14
Enrollment of Participants 14
Criteria for Taking a Participant Off Study 14
Measurement of Effect 15
Primary Outcome 15
Oral Glucose Tolerance Test (OGTT) 15
Secondary Outcomes 16
MR Spectroscopy 16

Hepatic fat spectroscopy 16
Hepatic fat quantification 17
Measurement of Inflammatory Cytokines and Hormones 17
DEXA Scan 18
Physical Activity and Nutrition Assessment 18
Muscle Performance and Physical Function 18
Energy and Sexual Function 18
Statistical Considerations 18
Design 18
Quantification of OGTT Outcomes (Specific Aim 1) 19
Quantification of Mechanistic Outcomes (Specific Aim 2) 19
Overall Analytic Approach 20
Aim-specific Analytic Plan 20
Specific Aim 1 20
Specific Aim 2 21
Additional exploratory analyses 22
Sensitivity Analysis and Missing Data 22
Statistical Computing, Data Security, and Data Sharing 22
Data Management Plan 22
Risks to the Subjects 23
Characteristics of the Patient Population 23
Sources of Materials 23
Potential Risks 23
Adequacy of Protection against Risk 25
Recruitment and Informed Consent 25
Protection against Risk 25
Potential Benefits to the Subjects 25
REFERENCES 26

Rationale and Objectives

Introduction

Prostate cancer (PCa) is the most common cancer in men in the United States. As testosterone has a central role in the stimulation of prostate tissue, androgen deprivation therapy (ADT) is the cornerstone of treatment in men with high grade and metastatic PCa. ADT results in castrate levels of serum testosterone and this profound androgen deficiency leads to an increase in both visceral and subcutaneous fat mass, and a reduction in skeletal muscle mass. These unfavorable changes in body composition result in the development of insulin resistance and cardiovascular disease. These metabolic perturbations follow an aggressive course in these men. These adverse metabolic changes have significant consequences as men on ADT have a higher risk of cardiovascular disease including coronary artery disease, myocardial infarction, peripheral vascular disease and sudden cardiac death compared with those men with PCa who do not undergo ADT and are only treated with prostatectomy. Indeed, cardiovascular disease has become the leading cause of mortality in these men. Although insulin resistance is the seminal event in this metabolic cascade, the predominant site and the mechanisms behind its development (such as the role of parenchymal fat infiltration and inflammatory cytokines) remain unknown in this patient population. Unveiling the site and the mechanisms of this resistance will guide physicians to prevent insulin resistance by initiating "tissue-specific" insulin sensitizing drugs in the future.

Our **overall objective** is to conduct a prospective, 6-month, observational cohort study to **1)** determine the predominant site of insulin resistance by using a validated, state-of-the-art, oral glucose tolerance test, and **2)** determine the mechanism of insulin resistance by measuring hepatic and intra-myocellular fat (using magnetic resonance spectroscopy) and measuring circulating inflammatory cytokines in non-diabetic men who are about to undergo ADT for their PCa (**ADT group**). We will also enroll a control group of non-diabetic men who had undergone prostatectomy for localized PCa (and are deemed cured) and never underwent ADT. Our **first aim** is to determine the site of insulin resistance (liver or muscle) in men undergoing ADT using a validated, state-of-the-art, oral glucose tolerance test. Our **second aim** is to determine mechanisms behind insulin resistance by measuring circulating inflammatory cytokines and measuring hepatic and intra-myocellular fat by magnetic resonance spectroscopy in both cohorts.

Study Design

This will be a prospective, single-center, 24-week, observational cohort study in men, 19 years and older, who are about to undergo ADT (**ADT-group**) and compare it over time with a control group of men with PCa who have undergone prostatectomy in the past and have not undergone ADT (**non-ADT group**). Men in the ADT-group must be hormone-naïve [first time receiving ADT] and have planned ADT for at least 24-weeks. Both cohorts (ADT-group and Non-ADT group) will be non-diabetic and without any prior history of physician-diagnosed hypogonadism. To achieve our objectives, we will enroll **50** participants in the ADT-group and **25** participants in the non-ADT group (**N=75 participants overall)**. This target sample of 75 participants will have >90% power to test our primary hypothesis.

Primary Objective

Our primary objective is to determine the **predominant site of insulin resistance** in men undergoing ADT by performing state-of-the-art OGTT that will determine whether the insulin resistance is at the level of the liver or skeletal muscle. This OGTT will be performed in hormone-naïve, non-diabetic men who are about to undergo ADT (**ADT-group**) for treatment of their PCa by their oncologists and also in a control group of non-diabetic men with prior history of PCa

who have undergone prostatectomy in the past, are deemed cured and have never received ADT (**non-ADT group**). The **ADT-group** will undergo OGTT at baseline, 12-weeks and 24-weeks into castration while the **non-ADT** group will undergo OGTT at the same time-intervals from time of enrollment.

Secondary Objectives

We have the following secondary objectives:

- To correlate the development of insulin resistance with deposition of intra-myocellular fat and hepatic fat by using non-contrast MR spectroscopy[115].
- To determine the role of inflammatory cytokines in the development of insulin resistance.
- To correlate the changes in body composition with the development of insulin resistance.

Exploratory Objectives

We have the following exploratory objectives:

- To assess the changes in muscle strength in the two groups with leg press strength and power test.
- To assess the changes in physical function in the two groups with loaded stair climb, power and 6-minute walk tests.

Background

Prostate cancer (PCa) is the most common cancer in men in the United States. Estimates suggest that in 2017, 161,360 new cases were diagnosed in the United States alone, with deaths occurring in 26,730 men[1]. Prostate is an androgen-dependent organ. In 1941, Huggins and Hodges described its androgen-dependence by showing that orchiectomy retards the growth of metastatic PCa[2,48]. Seven decades later, ADT remains a cornerstone of treatment in men with locally advanced, recurrent and metastatic PCa and has shown improved survival in these patients. Current estimates suggest that approximately 600,000 men in the United States alone are receiving ADT[3-5]. The aim of ADT is to achieve serum testosterone levels in the castrate range i.e.<50 ng/dl (normal male levels 300-1000 ng/dl)[49]. The methods of ADT include orchiectomy or medical therapy (gonadotropin releasing hormone [GnRH] agonists and antagonists), with most men choosing medical therapy[50]. In addition to men with locally advanced and metastatic PCa, there has been an increasing use of ADT even in patients with early stage PCa and those experiencing biochemical recurrence (rising PSA) after an initial period of remission after prostatectomy[51-53]. As a result, the use of ADT in the United States has increased 10-fold[54] resulting in a large number of men experiencing profound androgen deficiency and predisposing them to developing insulin resistance and CVD[55-57].

The profound androgen deficiency that occurs as a consequence of ADT not only leads to sexual dysfunction, hot flashes and low bone mass[6], but also results in a significant increase in fat mass (both subcutaneous and “metabolically active” visceral fat) and loss of muscle mass[7,8]. These unfavorable changes in body composition are the seminal events that lead to development of insulin resistance, an independent risk factor for atherosclerosis and cardiovascular disease (CVD)[9-12] and also a precursor to diabetes and metabolic syndrome[13-15].We have reported that increase in fat mass(both abdominal and extremities) in men

undergoing ADT is associated with severe insulin resistance, diabetes and metabolic syndrome[16-20]. Interestingly, these metabolic conditions display an accelerated course in androgen-deprived men as insulin resistance develops within 6-8 weeks of initiation of ADT[21-23]. These metabolic perturbations promote development of CVD. We have also reported that men undergoing ADT have significantly higher rate of incident coronary artery disease, myocardial infarction, peripheral vascular disease and cardiovascular mortality when compared with men not receiving ADT[24,25]. In fact, CVD is a common cause of mortality in men with PCa[26,27].

Factors Predisposing Men undergoing ADT to develop Metabolic Changes:

Changes in Body Composition

Preclinical data show that androgens promote differentiation of pluripotent uncommitted mesenchymal stem cells towards myogenic lineage and inhibit their commitment towards adipogenic lineage[58-60]. Deprivation of androgens results in the opposite effect. It is well established that androgen deficiency in men with pituitary and testicular disease is associated with an increase in fat mass and a decrease in muscle mass[61,62]. Experimental suppression of testosterone by GnRH agonists even in healthy young men reduces muscle mass and increases fat mass[63]. Similarly, age-related decline in testosterone is also associated with this phenotype[64]and testosterone replacement in hypogonadal men increases muscle mass and reduces fat mass[65-68]. Similarly, men undergoing ADT experience unfavorable changes in body composition that play a key role in the pathogenesis of metabolic complications[4]. This increase in fat mass involves both subcutaneous and visceral fat, the latter being metabolically active[69]. We previously reported that men undergoing ADT have greater abdominal obesity compared with eugonadal men with PCa not undergoing ADT (non-ADT) and eugonadal age-matched non-cancer controls[7,16-19]. This change in body composition follows an accelerated course as the increase in fat mass (2 kg) and decrease in muscle mass (1.7 kg) are evident within 6-8 weeks of ADT[21,70,71]. On average, these men experience an increase in BMI by 2.4% and in fat mass by 9.4%,while lean mass is reduced by 2.7%, confirming that this increase in BMI is predominantly due of accumulation of fat mass[21,70,71]. These studies demonstrate that ADT results in an unfavorable body composition and the increase in fat mass correlates positively with insulin resistance[21].

Inflammation

Testosterone possesses immunosuppressive properties as there is greater predisposition of women and androgen-deficient men to autoimmune diseases[95]. Addition of testosterone to cell culture attenuates production of inflammatory cytokines and testosterone replacement in castrated male mice suppresses production of TNF-α[96]. Human studies also confirm inverse relationship between cytokines and testosterone. A large population study of 467 men that testosterone levels were negatively correlated to soluble IL-6 receptor[97]. Experimental hypogonadism in men induced by GnRH agonists also show a significant increase in IL-6 and TNF-α[98]. Interventional studies show that testosterone replacement in hypogonadal men suppresses TNF-α and IL-1β, while increasing IL-10 (anti-inflammatory cytokine)[99]. Since inflammation is a risk factor for insulin resistance[36,38], we believe that men undergoing ADT develop a hypogonadism-induced pro-inflammatory state, contributing to metabolic complications. Inflammation leads to insulin resistance via several mechanisms. Cytokines such as MCP-1, IL-1, TNF, and IL-6, are associated with the progression from normoglycemia to insulin resistance[100,101]. TNF-α and IL-6antagonize the action of insulin at the receptor level. Cytokines released from adipose tissue produce endocrine effects on distant organs, such as muscle and liver, exacerbating insulin resistance[102]. In abdominal obesity, macrophage infiltration occurs in the visceral adipose tissue, which negatively impacts insulin sensitivity by secreting free fatty acids and adipokines. The role of cytokines in insulin resistance is further

confirmed by observations that neutralization of TNF-α improves insulin sensitivity[103]. We plan to measure these markers of inflammation in our study.

Deposition of Hepatic and Intramyocellular Fat

Lipotoxicity is an important contributing factor to the metabolic derangements in obesity and leads to insulin resistance. Lipids result in insulin resistance by a) free fatty acids released from visceral fat that interfere with insulin action[89], b) fat metabolism leading to the accumulation of intracellular lipid products[90]. This lipid accumulation mainly occurs in muscle and liver[91]. In general, excess fat is initially stored in adipose tissue. However; in abdominal obesity, the capacity of adipose tissue storage is exceeded and the excess fat “spills over” into skeletal muscle and liver[92]. This tissue lipid accumulation can also serve as a biomarker for the insulin-resistant state[93,94]. However, this remains unexplored in men undergoing ADT.

CVD and Mortality

Metabolic perturbations resulting from ADT are associated with increased CVD and mortality. The last decade has shown that deaths due to CVD have become the leading cause of mortality in men with PCa[26,27]. Men undergoing ADT have a higher risk of incident coronary artery disease, myocardial infarction and sudden death compared with non-ADT men[24].These men also have a 20% higher risk of CV morbidity than men not receiving ADT[83]. Androgen-deprived men also have shorter times to fatal myocardial infarction[84,85]and atherosclerosis related congestive heart failure[86]. As insulin resistance is the promoter of atherosclerosis, determining the site and mechanisms of this resistance is the key to preventing CVD.

Rationale

Although the development of ADT-induced insulin resistance is well-established, the predominant site of insulin resistance (muscle or liver) remains unclear. Systemic insulin resistance is a sum of insulin resistance that occurs in different organs, predominantly the liver and the skeletal muscle[28-32]. This study will investigate the site of insulin resistance in men undergoing ADT by using a validated, state-of-the-art, oral glucose tolerance test (OGTT)[33]. What also remains unclear are the mechanisms that lead to ADT-induced insulin resistance. Evidence in the general population shows that men with insulin resistance have higher levels of inflammatory cytokines and fat infiltration of parenchymal organs, such as muscle and liver[34-41]. However, it remains to be determined if deposition of fat in these organs contribute to insulin resistance in men undergoing ADT. To determine that, we will measure hepatic and intramyocellular fat by using state-of-the-art magnetic resonance spectroscopy (MRS). We will also measure inflammatory cytokines and adipokines, that are known to induce insulin resistance in other cohorts, employing sensitive, validated assays. Unraveling of the site of insulin resistance will allow physicians to initiate targeted "tissue-specific" therapy with novel insulin sensitizing agents that affect insulin resistance at specific organs (liver or muscle), minimizing harm and maximizing benefit. Similarly, the role of inflammatory cytokines in the pathogenesis of insulin resistance in this population deserves exploration as newer drugs that antagonize action of these cytokines have shown to improve insulin resistance in other populations[42-44]. As men undergoing ADT are old and frail[45,46] with multiple co-morbidities, the option of physical exercise is limited, and there is a great need for targeted "tissue-specific" drug delivery. In addition to achieving our goals, the findings of this study will lay the groundwork for prospective intervention trials with "tissue-specific" insulin-sensitizing agents, novel anti-

inflammatory agents or Selective Androgen Receptor Modulators (SARMs)--agents that increase muscle mass and decrease fat mass while sparing the prostate[47], in the prevention of development of insulin resistance during ADT, which in turn will lessen the burden of CVD in this population.

Participant Selection

This is a prospective, single-center, 24-week, observational cohort study in men, 19 years and older, who are about to undergo ADT (ADT-group) and compare it over time with a control group of men with PCa who have undergone prostatectomy in the past and have not undergone ADT (non-ADT group). Men in the ADT-group must be hormone-naïve [first time receiving ADT] and have planned ADT for at least 24-weeks. Both cohorts (ADT-group and Non-ADT group) will be non-diabetic and without prior history of physician-diagnosed hypogonadism. To achieve our objectives, we will enroll 50 participants in the ADT-group and 25 participants in the non-ADT group (N=75 participants overall).

Eligibility Criteria

The following inclusion and exclusion criteria will be implemented. Some criteria are common that apply to both groups while some are specific to the individual groups.

Inclusion Criteria for ADT-group:

- Hormone-naïve men about to undergo surgical or medical ADT
- Planned ADT duration (per their oncologists) for at least 24-weeks
- No known history of (physician-diagnosed) diabetes
- No known history of (physician-diagnosed) hypogonadism

Inclusion Criteria for Non-ADT Patients:

- PCa survivors who have undergone radical prostatectomy and are in remission
- Have never received ADT
- No known history of (physician-diagnosed) diabetes
- No known history of (physician-diagnosed) hypogonadism

Common Exclusion Criteria for both Cohorts:

- Any prior history of hypogonadism (known hypothalamic, pituitary or testicular disease)
- History of diabetes (physician-diagnosed) or on any diabetic medications
- Fasting glucose ≥126 mg/dl or HbA1c>6.5%[112]
- Use of systemic glucocorticoids (consecutive use for 2-weeks) within the past 3 months
- Use of opioid analgesics (consecutive use for 2-weeks) within the past 3 months
- Contraindications to magnetic resonance spectroscopy (MRS) study (some participants who cannot undergo MRS but can undergo other study procedures might be considered on a case-by-case basis). These include:
  a) *electrical implants such as cardiac pacemakers*
  b) *ferromagnetic implants such as aneurysm clips, surgical clips, artificial hearts valves with steel parts, shrapnel or steel implants*
  c) *ferromagnetic objects such as jewelry or metal clips in clothing*
  d) *pre-existing history of claustrophobic reactions*

Rationale for the Study Duration

The typical duration of ADT in patients with PCa is 24-weeks. We will follow the subjects throughout the course of their 24-week treatment duration. As previous studies have shown that insulin resistance develops within 6-8 weeks of initiation of ADT[21,22], the 24-week follow-up duration will assuredly allow us to accomplish our objectives.

Schedule of Study Procedures

A schedule of study procedures is provided below.

Procedures	Screening	Baseline	12-Weeks	24-Weeks
Eligibility/ Consent	X			
Medical History	X			
**Procedures**	**Screening**	**Baseline**	**12-Weeks**	**24-Weeks**
Vital Signs, EKG and Anthropometry (BMI, waist/hip ratio)	X	X	X	X
Screening Labs: Fasting glucose, HbA1c, Testosterone	X			
Physical Exam	X			
Oral Glucose Tolerance Test		X	X	X
MR Spectroscopy (liver and gastrocnemius muscle)		X		X
Body Composition (DEXA Scan)		X	X	X
Total and Free Testosterone, SHBG, Luteinizing Hormone		X	X	X
Inflammatory Cytokines (CRP, TNF-α, IL-1β, IL-6), Adipokines (leptin, adiponectin, resistin), Free fatty acids		X	X	X
CBC, CMP, PSA		X	X	X
Fasting Lipid Profile, Fasting Glucose, HbA1c		X	X	X
Questionnaires (RAPA, HWFF, HED, SAID, PF-10)		X	X	X
Strength and Physical Function Assessments (leg press strength and power/6-min walk/loaded stair climb power)		X	X	X

Screening, Recruitment and Enrollment

Recruitment of ADT-group from Oncology Clinics at the Dana-Farber Cancer Institute (DFCI)

The Dana-Farber Cancer Institute was incorporated in Massachusetts in 1951 as a non-profit institution engaged in cancer research and treatment. The DFCI is strictly an outpatient facility. Approximately 15,000 new outpatients are seen at the DFCI per year. DFCI is one of the leading centers in the evaluation and treatment of patients with PCa. Every year, approximately 1200 new patients with PCa are seen and ~100 PCa patients are put on therapeutic clinical trials and ~900 are placed on non-therapeutic trials including cohort studies, specimen banking studies and genotyping studies. The Lank Center for Genitourinary Oncology provides the umbrella for clinical and research activities in PCa. Each year, approximately 250 new patients initiate ADT for PCa in the oncology department. Dr. Taplin is a world-renowned PCa researcher and is the Director of the Lank Center for Genitourinary Oncology and has recruited

a large number of patients with PCa in both single-center and multi-center trials. Similarly, Dr. Paul Nguyen is an internationally known oncologist focused exclusively on the care of men with PCa and is the Director of the Prostate Brachytherapy program. Drs. Taplin and Nguyen (and their colleagues) evaluate more than 550 men a year with a new diagnosis of prostate cancer. Recruitment efforts will be spearheaded by Dr. Basaria and his staff, who has experience in recruiting subjects for previous ADT studies and has successfully recruited both ADT and non-ADT patients from Oncology and Urology clinics at the DFCI and BWH.

The following will be our additional efforts for targeted recruitment of ADT-group:

1. The study coordinator will engage with Dr. Taplin's and Nguyen's coordinators, nurses and physician assistants to identify patients that are planning to undergo ADT. These patients will then be approached in "real time" by the investigators and the study will be introduced to these patients. If a patient is interested in participating, the study coordinators will approach the patient and will be given an appointment for the screening visit which will take place at Dr. Basaria's research clinic at BWH. The above-mentioned process has been successfully implemented in other studies in which Drs. Basaria, Taplin and Nguyen have collaborated.

2. Study flyers will be posted in oncology clinics at the DFCI.

3. Study flyers will also be posted in the oncology satellite clinics outside the DFCI that are under the Partners health care system. Drs. Basaria, Taplin and Nguyen will reach out to oncologists at these clinics and seek their assistance.

4. Drs. Basaria, Taplin and Nguyen will also reach out to oncologists at other hospitals under the Partners Health Care System and seek their collaboration. These hospitals include Massachusetts General Hospital, Newton-Wellesley Hospital and Faulkner Hospital.

5. Advertisements will be placed in the local periodicals and newspapers which will enhance recruitment by introducing the study to PCa patients that are seeking care at the other medical centers in Boston and wider New England region.

6. Study brochures will be placed in and handed out at the DFCI Oncology clinic and BWH Urology clinic, which includes at satellite locations. Also, study brochures may be handed out at prostate cancer support groups or at other various clinics.

Recruitment of Non-ADT group from Urology Clinics at Brigham and Women's Hospital
The Division of Urology is well known internationally and Dr. Adam Kibel, a collaborator on this proposal, is its Chief. Approximately 300 prostatectomies are performed each year in the Division of Urology, creating a large pool of men who are potential candidates for our control (non-ADT) group. Dr. Kibel's clinic nurses and research assistants will facilitate Dr. Basaria's team to recruit patients. The Urology Division has prospectively enrolled a large number of post-prostatectomy patients in numerous studies. In addition, existing databases within the Division of Urology have over 10,000 patients who have previously undergone prostate surgery at BWH. Dr. Basaria's previous studies in the non-ADT population have successfully enrolled non-ADT patients as the database of these post-surgical patients exceeds 10,000. The urology clinic has numerous active clinical trials and research programs and has a dedicated space that is adjacent to the clinical offices for facilitating patient enrollment.

The following will be additional measures for targeted recruitment of the Non-ADT group:

1. Dr. Kibel's team will provide Dr. Basaria's team the list of the patients in the database who have undergone prostatectomies and have given permission to be contacted for a research study. Once the list is obtained, the dedicated study coordinator will call these potential participants. If the subject is interested, the participant will be screened over the phone and if eligible, will be asked to come for an in-person screening visit at the research clinic.

2. The study coordinator will also regularly connect with Dr. Kibel's coordinators and physician assistants to identify patients who are coming to the clinic after their prostatectomy. These patients will then be approached in "real time" by Dr. Kibel during their clinic visit and the study will be introduced to these patients. If the patient is interested in participating, the study coordinator will approach the patient. An interested participant will be given an appointment for an in-person screening visit at the research clinic.

3. Study flyers will be posted in Urology clinics at the BWH.

4. Study flyers will also be posted in the Urology satellite clinics that are under the Partners health care system. Dr. Kibel will reach out to urologists at these clinics and seek their collaboration.

5. Advertisements will be placed in the local periodicals and newspapers which will enhance recruitment by introducing the study to PCa patients that are seeking care at the other medical centers in Boston and wider New England region.

6. A detailed post advertising the study will be put on https://clinicaltrials.partners.org/ where interested individuals can sign up to be contacted about the research study.

Inclusion of Minorities
Persons of all racial groups will have an equal chance of participating in the study. As the prevalence of PCa is greater in African-Americans and there is a great unmet need to study metabolic complications in them, we will oversample African-American men which will comprise 40% of the sample. Overall, 53% of our sample now comprises diverse group of minorities (**Enrollment Table**).

**Racial Categories**	**Ethnic Categories**									**Total**
	**Not Hispanic or Latino**			**Hispanic or Latino**			**Unknown/Not Reported Ethnicity**			
	**Female**	**Male**	**Unknown/ Not Reported**	**Female**	**Male**	**Unknown/ Not Reported**	**Female**	**Male**	**Unknown/ Not Reported**	
American Indian/ Alaska Native	0	3	0	0	0	0	0	0	0	3
Asian	0	6	0	0	0	0	0	0	0	6
Native Hawaiian or Other Pacific Islander	0	1	0	0	0	0	0	0	0	1
Black or African American		25	0		5		0	0	0	30
White		30	0		5	0	0	0	0	35
More Than One Race			0	0	0	0	0	0	0	0
Unknown or Not Reported		0	0	0	0	0	0	0	0	0
**Total**	0	65	0	0	10	0	0	0	0	75

This is a prospective observational cohort study of 24-weeks duration in men who are about to commence ADT from their oncologists as part of their PCa treatment (**ADT-group**) and in men who have previously undergone radical prostatectomy for localized PCa (**Non-ADT group**). There is ***no drug administration involved in this study***. Only study procedures (OGTT, DEXA scan, MRS, etc.) will be performed that are relevant to the evaluation of study outcomes.

Enrollment of Participants
Institutions will register eligible participants in the Clinical Trials Management System (CTMS) OnCore as required by DF/HCC SOP REGIST-101.

Criteria for Taking a Participant Off Study
Participants will be removed from study when any of the following might occur:

- Lost to follow-up
- Withdrawal of consent
- Death

Measurement of Effect

Primary Outcome
The primary outcome is to determine the predominant site of insulin resistance in men undergoing ADT by performing state-of-the-art OGTT that will determine whether the insulin resistance develops at the level of the liver or skeletal muscle. The detailed methodology of this OGTT is summarized below.

Oral Glucose Tolerance Test (OGTT)

Pharmacological intervention with insulin-sensitizing drugs not only improves insulin sensitivity but also reduces the risk of diabetes[116,117] and CVD[118,119]. Systemic insulin resistance is a sum of insulin resistance that develops in different organs, particularly the liver and the skeletal muscle. Since novel pharmacological interventions that improve insulin resistance at specific organs are being developed, quantifying the magnitude of insulin resistance in these organs deserves exploration. Matsuda and DeFronzo have developed a state-of-the-art OGTT to quantify "tissue-specific" insulin resistance in a safe and timely manner. This technique is as accurate as clamps, but less burdensome than the insulin-glucose clamp technique[110]. We will perform this OGTT in our participants. The procedure entails administration of 75 g of glucose. Blood samples for glucose and insulin will be collected at −30, −15, 0, 30, 60, 90, and 120 min. In the post-absorptive state, the higher the rate of endogenous glucose production and higher the fasting plasma insulin concentration, the greater the severity of hepatic insulin resistance. As 85% of endogenous glucose is produced in the liver[120], at baseline, endogenous glucose production primarily reflects hepatic glucose production. The product of endogenous glucose production and fasting plasma insulin, therefore, provides a measure of hepatic insulin resistance under post-absorptive conditions[121,122]. Following the glucose load, the rise in plasma glucose concentration stimulates insulin secretion from the pancreas and the resulting hyperinsulinemia suppresses endogenous glucose production. In subjects with normal hepatic insulin sensitivity, this rise in plasma glucose and insulin is sufficient to suppress endogenous glucose production[121,122]. However; in individuals with hepatic insulin resistance, the suppression of endogenous glucose production is at best modest, thereby causing a greater increase in plasma glucose during the early phase (0–30 min) of OGTT. The magnitude of the rise in plasma glucose and insulin concentrations 0–30 min after glucose load is proportional to the magnitude of hepatic insulin resistance. The total AUC during the OGTT reflects the combination of the fasting and post-absorptive plasma glucose and insulin concentrations, and the product of glucose AUC and insulin AUC provides an index of hepatic insulin resistance (hepatic insulin sensitivity index).

As there is no significant change in the rate of endogenous glucose production during the 60- to 120-min time period of the OGTT, the decline in plasma glucose concentration after 60 min primarily reflects glucose uptake by skeletal muscle. Therefore, the decline from the peak plasma glucose concentration during the OGTT is determined by the combination of two factors:*1*) skeletal muscle insulin resistance and *2*) plasma insulin concentration. The greater the muscle insulin resistance and the lower the plasma insulin concentration, the slower is the decline in plasma glucose concentration. Thus, skeletal muscle insulin sensitivity is calculated as follows: Muscle insulin sensitivity index = dG/dt ÷ mean plasma insulin concentration (*I*), where dG/dt is the rate of decline in plasma glucose concentration. Hence, this OGTT provides an index of whole-body as well as organ-specific insulin sensitivity such as the product of AUC for glucose and insulin during the first 30 min of the OGTT ($\text{glucose}_{0\text{–}30}[\text{AUC}] \times \text{insulin}_{0\text{–}30}[\text{AUC}]$)

strongly correlate with the hepatic insulin resistance ($P < 0.0001$) while the rate of decay of plasma glucose concentration from its peak value to its nadir during the OGTT divided by the mean plasma insulin concentration (dG/dt / I) strongly correlates with muscle insulin sensitivity ($P < 0.0001$)[33]. Hence, the initial rate of rise in plasma glucose concentration is mainly determined by hepatic insulin resistance while the rate of decline in plasma glucose concentration from its peak value to its nadir reflects muscle insulin resistance.

Secondary Outcomes

The secondary outcomes include the following:

- To correlate the development of insulin resistance with deposition of intra-myocellular fat and hepatic fat by using MR spectroscopy[115].
- To determine the role of inflammatory cytokines in the development of insulin resistance.
- To correlate the changes in body composition with the development of insulin resistance.

The detailed methodology to evaluate these outcomes are summarized below.

MR Spectroscopy

All MRI examinations will be performed using a 3-Tesla clinical imaging unit. Subjects will be imaged with the mixed fast spin-echo pulse sequence that combines T1 and T2 weighting in a single acquisition[123]. The quantitative MR imaging distributions will be volumetric[124]. The images will be transferred in a Digital Imaging and Communications in Medicine format to image-processing laboratory for further analysis. The data sets will then be entered into computer programs developed using MathCad 2001i software (Mathsoft, Cambridge, Mass) to evaluate spatial anatomic data, segment the organs and calculate the volumes.

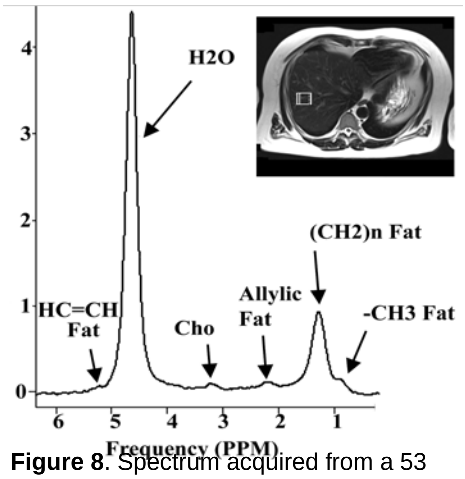

Figure 8. Spectrum acquired from a 53 year old male with a healthy liver. Lipid resonances are indicated.

Hepatic fat spectroscopy

Localized proton MR imaging and spectra of the liver will be acquired with subjects in the supine position with a 3-Tesla MRI scanner (Siemens AG, Erlangen, Germany) using body coil and a torso-matrix coil. Steady-state free precession imaging will be acquired in the axial and coronal planes in order to delineate blood vessels and biliary system followed by an axial single shot turbo spin echo to delineate bile ducts. These images will be used to localize the MRS voxel. Two areas of interest will be selected to quantitate hepatic fat (avoiding vasculature and non-hepatic structures) using universal body point resolved spectroscopy in two regions of the hepatic lobe with volume of 30x30x30 mm and 64 averages using 20 second breath holds[125-127]. Frequency domain chemical shift correction will be applied to eliminate movement artifacts. Data will be doubly zero-filled, phased, and a line broadening of 8 Hz applied after Fourier transform as shown in **Figure 8**.

Hepatic fat quantification

Spectra will be exported offline for post-processing. Intensities of the peaks resonating form the protons of hepatic water (4.7 ppm) and protons of methylene groups (-CH2-) in the fatty acid chains (1.3 ppm) will be determined, corrected for T1 and T2 relaxation, and liver fat content will be expressed as a ratio of signal of the methylene group to total signal of methylene plus water. Hepatic lipid content will be determined from the relative percentage of spectral peak at 1.3 ppm (representing the sum of triglyceride acyl chains) over the spectral peak of protons from hepatic water at 4.7 ppm. To convert the relative quantity of hepatic fat and water into absolute concentrations, we will use the method described by Szczepaniak et al[127]. This method has been widely accepted as the most accurate assessment of liver fat content[127]. Proton MR spectroscopy will provide qualitative information on liver lipids that predict the degree of lipotoxicity. The total amount of unsaturated fatty acids will be expressed as the resonance of olefinic protons relative to hepatic water. The polyunsaturation index will be expressed as percentage[128-130].

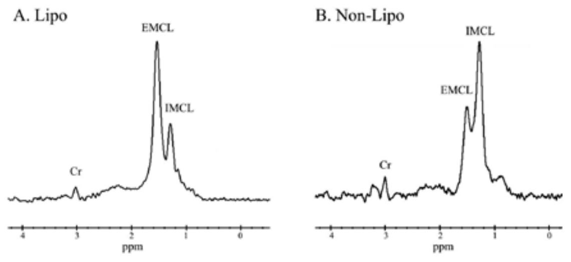

Figure 9. ^{1}H MRS of the tibialis muscle in A) lipodystrophy, B) without lipodystrophy. The results show a significantly increased EMCL as a result of the lipid-modifying condition.

Muscle fat spectroscopy

Taking advantage of the Total Imaging Matrix capability of the 3-T MRI scanner, *the muscle fat spectroscopy will be acquired immediately after the liver scan*. At the time of subject setup, an 8-cm surface coil will be fixed to the calf along the gastrocnemius muscle using Velcro straps. The patient will lie supine, feet first and place his foot in the plantar exercise device. Special care will be taken to ensure that the subject's leg is parallel with the bore to ensure optimal separation of the intramyocellular lipid content (IMCL) and extramyocellular lipid content(EMCL) resonances that is dependent on the isotropy of the muscle fibers along the B_0 field[131]. ^{1}H MRS single voxel spectra will be acquired from the gastrocnemius muscle (2x2x2 cm^3) for IMCL/EMCL analysis as shown in **Figure 9.**

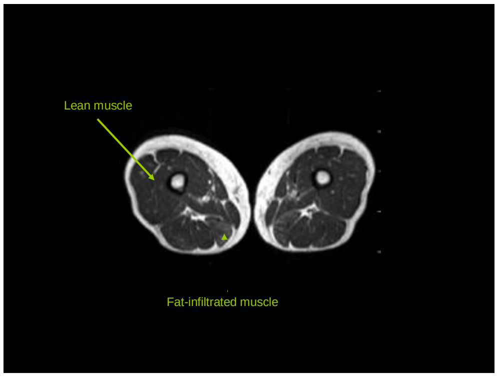

Figure 10. Axial section of the thigh with lean and fat-infiltrated muscle.

Muscle fat quantitation

^{1}H MRS single voxel PRESS scans will be processing using an automated time-domain based spectral fitting software (LC Model v6.3, Provencer) which will output concentrations of IMCL, EMCL, and Cr. **Figure 10,** shows axial section of the thigh illustrating lean and fat-infiltrated muscle.

Measurement of Inflammatory Cytokines and Hormones

The proposed analyses will be performed at Brigham Research Assay Core (BRAC), Mayo Clinic Core Lab and other research laboratories. Measurements will be performed on frozen plasma and serum banked in a -80º C freezer. Plasma glucose will be quantified using Beckman Glucose Analyzer. Serum insulin, leptin, resistin, TNF-α, IL-1β and IL-6 levels will all be measured using high sensitivity sandwich ELISA. Adiponectin will be measured using RIA.

C-Reactive Protein will be measured using a high sensitivity sandwich ELISA. Total

testosterone will be measured by using liquid chromatography tandem mass spectrometry (LC-MS/MS) with sensitivity of 2 ng/dL[132,133]. Sex Hormone Binding Globulin will be measured by solid-phase fluoroimmunometric assay. Free testosterone will be measured with equilibrium dialysis[68,134].

DEXA Scan

We will assess lean body mass and fat mass by dual-energy x-ray absorptiometry (DEXA) scan (Hologic QDR 4500A). The scanner will be regularly calibrated using a soft-tissue phantom. We have used these measures in many previous studies[7,16-19,47,68].

Physical Activity and Nutrition Assessment

We will assess physical activity utilizing the validated *Rapid Assessment of Physical Activity (RAPA)* questionnaire; a one-page tool that has been used in many clinical trials. We have previously used it in a NIA-funded trial (**NCT01275365)**[135,136] that assessed physical activity in older frail men. We will assess nutrition intake using *Harvard Willett Food Frequency Questionnaire* that has been extensively validated in many populations[137-139]. These assessments will be performed in both cohorts at baseline, 3 and 6 months and will be considered in sensitivity analyses.

Muscle Performance and Physical Function

We will assess physical function by measuring walking speed in the 6-min walk, stair climbing power, and self-reported physical function by the PF10 (physical function domain of MOS SF-36). Maximal voluntary strength and power in the leg will be assessed with the 1-RM method. These measures in many previous studies have been shown to be androgen-responsive and it would be very meaningful to assess the impact of ADT on these measures. These assessments will be performed in both cohorts at baseline, 3 and 6 months.

Energy and Sexual Function

We will assess energy/vitality by administering the validated *Hypogonadism Energy Diary* (*HED*) questionnaire[146,147]. Sexual function will be assessed using the validated *Sexual Arousal, Interest and Drive* (*SAID*) scale[146,147]. These assessments will be performed in both cohorts at baseline, 3 and 6 months.

Statistical Considerations

Design

This is a single-center prospective cohort study with the overall goal of determining the site and mechanism of insulin resistance in androgen deprivation therapy (ADT) for treatment of prostate cancer (PCa). To address this aim, the study will follow hormone-naïve, non-diabetic men undergoing ADT from the beginning of their treatment and forward for 24-weeks; this cohort is referred to as the **ADT group**. A parallel cohort of men with PCa diagnosis and who have been treated with prostatectomy will also be followed (the **non-ADT group**), in order to provide control observations of outcomes against which the experience of men undergoing ADT may be contrasted. Major outcomes will be assessed at study baseline (study entry, prior to beginning of ADT in the ADT group) and at 12 and 24 weeks post-baseline.

The project's Specific Aims are:

- **Specific Aim 1**: To determine the **site (liver or muscle) of insulin resistance** in men undergoing ADT using a validated, state-of-the-art, OGTT.
- **Specific Aim 2**: To determine the **mechanism of insulin resistance** in men undergoing ADT by measuring circulating inflammatory cytokines, and hepatic and intra-myocellular fat.

Primary analyses will deal explicitly with differences between the ADT and non-ADT groups. In addition, secondary analyses that will partially fulfill Specific Aim 2 will consider ADT exposure and other factors that are specific to ADT (and will not occur in the non-ADT group). In order that these analyses are well-powered, we propose to enroll twice as many subjects in the ADT group as in the non-ADT group; see below.

Quantification of OGTT Outcomes (Specific Aim 1)

At baseline, 12 weeks and 24 weeks, differentiation between insulin resistance occurring at the liver or at the muscle will be obtained using a two-hour OGTT. Among subjects exhibiting insulin resistance at the liver or muscle, circulating glucose (G) concentrations are expected to follow roughly the pattern displayed in **Figure 11**. For participants manifesting resistance predominantly at the liver, glucose concentrations are expected to peak at roughly 30 minutes and then decline steadily. Meanwhile, for participants expressing resistance predominantly at the muscle, concentrations are expected to achieve a lower peak and to decline less rapidly. Hepatic insulin resistance is quantified according to lack of suppression of hepatic glucose output in the initial period following start of OGTT. Therefore, following Abdul-Ghani and colleagues[33], we will quantify hepatic resistance using **the product of area-under-the-curve (AUC) for insulin and glucose concentrations** during the first 30 minutes of OGTT ($\text{glucose}_{0\text{–}30}$[AUC] × $\text{insulin}_{0\text{–}30}$[AUC]) for each subject.
Level of insulin resistance at the level of the muscle is in rough (negative) proportion to the rate of decline of plasma glucose as time (t) progresses, **dG/dt**, during the period between glucose peak until reaching its nadir. We will therefore quantify muscle insulin resistance using estimated **dG/dt divided by the mean plasma insulin concentration (I)**, estimated by regression analysis at the subject level. Please see **Section D.1** for greater detail. For each of these, **change from baseline to 12 and 24 weeks** will be computed at the subject level, and analyses will be performed on the resulting change scores.

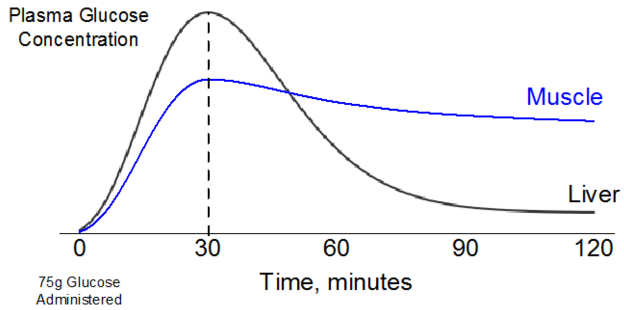

Figure 11. Anticipated pattern of change in plasma glucose concentrations for participants expressing insulin resistance primarily at the level of the liver ("Liver") and at the skeletal muscle ("Muscle"). For Specific Aim 1, analyses will focus on first 30 mins following start of OGTT to characterize hepatic resistance while time period from peak glucose concentration to nadir to characterize muscle insulin resistance.

Quantification of Mechanistic Outcomes (Specific Aim 2)

Concentrations of inflammatory cytokines (measured continuously) will be determined using methodology described above. Muscle and hepatic fat concentrations will be obtained from MRI (baseline and 24 weeks) and overall body composition assessment made available using DEXA (baseline, 12 and 24 weeks).

Overall Analytic Approach

Prior to formal analysis, variable distributions will be summarized by cohort (ADT and non-ADT groups). Means, standard deviations, ranges, medians, quartiles, and skewness will be determined. Graphical methods will be utilized to assess overall distributional properties. Outlying values will be noted and tracked for eventual consideration in sensitivity analyses (see below); likewise, potentially meaningful differences between arms will be noted and recorded for inclusion in propensity scoring (see below). Scatterplot smoothing and exploratory assessments via generalized additive models will be employed to assess the functional form of associations between covariates and outcomes. Confounding or effect modification by demographic factors such as age, race and other factors will be evaluated via graphical assessments and stratification. While statistical power to test for interaction by race will be limited, we will present exploratory evidence of interaction (or lack thereof) as context for reporting of results. Additional potential confounders and effect modifiers will include nutrition (measured the Willett FFQ) and physical activity (quantified by RAPA) which will be considered in sensitivity analyses. Following this, formal estimation of effects will be obtained using regression analyses as described below. All point estimates will be accompanied by 95% confidence intervals. Hypothesis tests will be evaluated at the 0.05 level. As hypotheses are pre-specified, no adjustment will be made for multiple comparisons.

Aim-specific Analytic Plan

Specific Aim 1 is concerned with determining the relative degree of insulin resistance at the level of the liver and at the skeletal muscle using, respectively, $\text{glucose}_{0–30}$[AUC] × $\text{insulin}_{0–30}$[AUC]) (denoted **AUC** hereafter) and **dG/dt / I**, as described above. These will be modeled separately. Each subject's change in AUC, dG/dt and I will be computed, and change at 12 and 24 weeks for each obtained. **Primary analyses** will estimate change at 12 and 24 weeks simultaneously using a mixed-effects regression analysis with control for cohort membership. Models will not control for baseline AUC, as this is generally thought to be inappropriate in non-randomized studies and may induce spurious correlations[140,141]. The differential increase in AUC with time accompanying ADT as compared to controls will be estimated using a treatment contrast and robust (employing sandwich variance estimators) 95% confidence interval, and quantification of statistical significance obtained using a likelihood ratio test. Analysis of dG/dt / I will proceed in like fashion. **Secondary analyses** will replicate these analyses controlling for participants' ages and baseline BMI as a simple index of body size, as well as other potential confounders as suggested in exploratory analyses described above. Where there is moderate evidence of effect modification, interaction terms will be incorporated in multiple regression analyses on the basis of consistency with clinical expectations, as hypothesis tests of interaction terms are likely to be underpowered and overly conservative[142].

Table 1. Minimum detectable difference between ADT and non-ADT groups, Specific Aim 1, assuming correlation 0.5 between repeated outcome measurements.

Statistical Power	**$\text{glucose}_{0–30}$[AUC] × $\text{insulin}_{0–30}$[AUC]**	**dG/dt / I**
95%	10.04	1.51
90%	9.03	1.35
85%	8.34	1.25
80%	7.80	1.17

The proposed **sample size** is intended to facilitate comparisons of the ADT to non-ADT group in **Specific Aims 1 and 2**, as well as providing the necessary precision to estimate change in outcomes in analyses of the ADT group alone in **Specific Aim 2**. Computations are presented under assumptions described**.** Based on data published by Abdul-Ghani et al[33] and our participant profiles, we anticipate that the cross-sectional standard deviations (SD) *s* of AUC and dG/dt / I will be less than 10 and 1.5 units, respectively. Assuming stable cross-sectional

SD with time, the SD of change in outcomes is given by $\sqrt{2(1-\rho\rho)}$, where $\rho\rho$ is the Pearson correlation between repeated outcomes measures, which we anticipate will be high. Under the relatively conservative assumption that $\rho\rho$ = 0.5, an evaluable sample size of 40 participants in the ADT group and 20 in the non-ADT group will provide 90% power to detect a mean between-group difference in AUC change and dG/dt / I change of 9.03 units and 1.35 units, respectively, and 80% power to detect differences of 7.8 units and 1.17 units (**Table 1**), respectively, at either follow-up time, differences of modest to moderate clinical significance based on their association with direct measures of insulin resistance[33]. Under these assumptions, a total of 40 evaluable subjects would in turn provide adequate precision to estimate mean change in AUC and dG/dt / I to within 3.2 units and 0.47 units, respectively, using 95% confidence intervals. Because the degree to which repeated measurements will increase statistical power is uncertain, we present these computations as conservative estimates; we anticipate that these computations underestimate to a modest degree the power obtained by the proposed design. Based on an assumption of cumulative attrition and missingness of 20%, we therefore propose a total sample size of 50 participants in the ADT group and 25 in the non-ADT group, or **N=75 participants overall**.

Specific Aim 2 is concerned with assessment of inflammatory markers and fat depots.

Analyses of inflammatory cytokines:
In **primary analyses**, unadjusted changes in inflammatory cytokines will be estimated using a mixed-effects model as described above, with between-group differences estimated using a treatment contrast and robust confidence interval. **Secondary analyses** will estimate within-subject changes in each cohort and as a function of baseline age, BMI and other covariates using generalized additive mixed models (**GAMM**)[143].

Analyses of hepatic and intra-myocellular fat:
These outcome measures will be obtained by MRS at baseline and 24 weeks. Change scores on each will be computed for each participant. In **primary analyses,** the mean within-subject change in fat measurements will be assessed, and between-group differences in change assessed using a point estimate and associated 95% confidence interval. In **secondary analyses**, the association between change and subject-level covariates including age, BMI, sex steroid levels, and lipids will be generated using a generalized additive model, which estimates trends and associated confidence regions using penalized likelihood. Where associations are sufficiently linear, multiple linear regression may be employed to generate parameters that are easily interpreted. We have successfully employed these approaches in obtaining estimates of nonlinear association in mechanistic models between sex hormone levels and functional & body composition outcomes in randomized clinical trials of older men[68].

As noted above, the **proposed sample size of 50 participants in the ADT group** (twice the size of the non-ADT group) is intended to facilitate evaluation of within-subject changes among participants exposed to ADT. Given projected attrition and missingness, we anticipate at least 40 evaluable change scores for any outcome and time point. Assuming, as above, relatively modest within-subject correlation of 0.5 in repeated measures of inflammatory cytokines or hepatic & intra-myocellular fat and cumulative missingness and attrition of 20%, this will provide adequate precision to estimate time-specific change in outcomes to within approximately 0.3 times the cross-sectional standard deviation of each measure using a 95% confidence interval within the ADT cohort. Under a conservative translation, this corresponds to an estimate of change in TNF-α to within approximately 4 pg/mL and of liver fat content to within 1.5%, a precision substantially subtler than the magnitude of the mean treatment effect on liver fat observed in a randomized clinical trial of pioglitazone in insulin resistance[126].

Additional exploratory analyses
Analyses paralleling those described above will be employed to assess differences in change in measures of physical capacity and function. Analyses of six-minute walk distance and average speed will assess between-group differences using mixed-effects regression models and propensity scoring as described above. We will perform similar analyses to assess between group differences in leg press strength and power. Previous analyses in similar populations have suggested that some measures of function may suffer from ceiling effects in relatively healthy older individuals. Accordingly, this study will also assess stair climbing power under a load, which our prior work has indicated may be more sensitive to change with time than unloaded stair climb power.

Sensitivity Analysis and Missing Data
We anticipate that outcomes will exhibit reasonable conformity with assumptions, but will assess distributional properties, observations' outlyingness, etc. as described above. In the case of outlying values, models will be re-estimated excluding these observations to ensure that qualitative conclusions are stable and robust. Where conformity with other modeling assumptions is threatened, robust alternatives such as analyses of rank-transformed change in outcome measures will be considered. Based on prior experience, we anticipate only a small number of missing records on subjects not lost to attrition. Analyses described above will obtain consistent estimates provided the missing data mechanism is noninformative. We will model the missing data process as a repeated-measures binary outcome using the modified (robust) Poisson regression model[144] as a function of study cohort and baseline covariates age, BMI, and testosterone concentration as well as study outcomes; results will be disclosed in publication. If appropriate, a pattern-mixture model[145] of outcomes and the missing data process will be estimated.

Statistical Computing, Data Security, and Data Sharing
The Biostatistical Design and Analysis Core of the Boston Pepper center (co-I Dr. Travison is the Core Leader) maintains computing resources on Microsoft Windows and Unix-based platforms, and software licenses to perform the analyses. Analyses will be performed using SAS version 9.4 (SAS Institute, Cary, NC) or R version 3.1 or later (R Foundation for Statistical Computing, Vienna). Simultaneous modeling of missing data and repeated-measures outcomes will be performed using MPlus version 7 or later (Muthén and Muthén, Los Angeles, CA) or equivalent software. Analytic datasets will be derived from databases maintained separately from subject identifiers; analysts will have no contact with study subjects or their identifying information. Following completion of the project, the lead biostatistician (Dr. Travison) will generate a data set suitable for data sharing purposes, which will be de-identified and stripped of protected health information (PHI) according to HIPPA and Safe Harbor principles.

Data Management Plan

The data management team will provide data management and programming support under the supervision of the statistician, Dr. Travison. The study will utilize a secure electronic data capture (EDC) system. The database will be designed to track subjects' current status, and to provide documentation of eligibility and consent, alerts concerning upcoming visit dates,

enrollment tracking, data capture, and real-time data validation. It will be hosted on secure, password-protected servers maintained by Partners Research Information Services and Computing (RC), which provides firewall, maintenance, data security, and backup services. Access to the database for entry and queries will be provided using a secure web portal. The data management facility at Brigham and Women's will act as the Data Coordinating Center (DCC), and will:

- Facilitate database design and quality control;
- Collaborate with CRF development;
- Develop standardized coding for electronic forms;
- Support electronic forms programming;
- Provide routine database testing;
- Insure data security and nightly backup;
- Maintain working relationships with the RC and investigators; and
- Provide all necessary services in support of data management and routine regulatory reporting.

The database will be fully HIPAA and 21 CFR part 11 compliant, providing full security and audit capabilities consistent with federal requirements and guidelines.

Risks to the Subjects

Characteristics of the Patient Population

The subjects are 50 patients with prostate cancer (PCa) who are about to undergo androgen deprivation therapy (ADT) and 25 patients with PCa who have undergone prostatectomy and are now in remission and have never received ADT. Patients with known history of diabetes or elevated fasting glucose/HbA1c levels during screening visit (based on American Diabetes Association guidelines) will be excluded. Furthermore, patients with known history of *testosterone deficiency* will also be excluded. The typical duration of ADT in patients with PCa is at least 6 months (24-weeks). Prior studies in this population have shown that insulin resistance develops within 6-8 weeks of starting ADT, suggesting that the 6-month observation period proposed will assuredly allow us to capture metabolic perturbations induced by ADT. Our analysis will assess treatment effects at 12-week (mid-point) and 24-week (end-of-study) time points.

Sources of Materials

Medical records of the subjects will be reviewed to determine the date of diagnosis, stage, grade and type of treatment given (or being given) for PCa. The patient's medical records may be reviewed with his consent to ascertain eligibility for this study.

Potential Risks

The potential risks of the study include the risks of blood drawing, dual energy X-ray absorptiometry, oral glucose tolerance test (OGTT), MR Spectroscopy and Questionnaires.

a) *Risks of Blood Drawing:* The risks of blood drawing include pain, bleeding and/or a bruise where the needle was inserted. Serious complications such as blood clots or infection are very rare when proper precautions are taken.

b) *Potential Risks of DEXA Scan:* The radiation exposure during a DEXA scan is minimal (less than 25 mrems, less than that from a chest X ray). While we do not know whether any dose of radiation is completely safe, this amount of radiation to which the subjects will be exposed during the course of the study is well within the limits considered "safe" by federal and state regulations.

c) *Potential Risks of OGTT*: The OGTT is a very safe procedure that has been used in a variety of research settings in various patient populations, including older subjects with co-morbidities. Unlike the euglycemic hyperinsulinemic clamps that are invasive and burdensome (including risk of hypoglycemia and hypovolemia), which is particularly relevant in our target population of older and frail men with multiple co-morbidities, the state-of-the-art OGTT that will be used in this study to calculate the Matsuda Index is not only comparable to the clamps in terms of assessing outcomes, but substantially safer compared to clamps.

d) *MR Spectroscopy Risks:* FDA considers investigations of MRI and MRS software and hardware operating within FDA specific parameters as non-significant risk device studies. All studies performed for this study will adhere to these (non-significant risk) FDA approved safety levels. These safety parameters include static magnetic field, time varying magnetic fields (dB/dt), specific absorption rate (SAR) and acoustic noise levels. Since MRS does not involve the use of radiation or other harmful material, it is safe to repeat imaging with no known long-term effects. Established risks for MRS include claustrophobia due to confinement of the patient in the system, the malfunction of electromagnetic implants, pregnancy, slight hearing impairment due to high acoustic noise levels and slight neuromuscular twitching (for the higher field strength systems). However, system safeguards and screening procedures have been designed and operating guidelines have been provided to minimize any of the aforementioned risks. Patient comfort will be verified periodically as described above. Subjects will be provided mandatory hearing protection (earplugs), which will prevent discomfort due to scanner noise. Given our careful screening procedures and the potential benefits outlined in the section on the significance of this research, we believe there is much knowledge to be gained at no significant risk to participants.

e) *Questionnaires:* We will administer validated questionnaires to assess physical activity and nutrition intake. Physical activity will be assessed by utilizing the Rapid Assessment of Physical Activity (RAPA) questionnaire which is a one-page questionnaire and has been used in many clinical trials. Both Drs. Basaria and Travison have recently completed a complex NIA-funded research (**NCT01275365, PMID: 28483498**) using RAPA questionnaire to assess physical activity in older frail men. Hence, the investigators have significant expertise in the use of this tool. We will also administer validated Harvard Willett Food Frequency Questionnaire to assess nutrition intake in our participants. These questionnaires have been used in a variety of study populations, are brief and simple; hence not likely to pose significant burden on the participants.

f) *Physical Function and Muscle Strength:* We have administered these tests to older men and women without any adverse events. Drs. Basaria and Storer have used these assessments in a complex NIA-funded research (**NCT01275365, PMID: 28483498**) to

assess physical activity in older frail men successfully. These assessments are also being used currently in another NIH-funded trial of SARMs in prostate cancer survivors without any side effects.

Exclusion criteria for MR Spectroscopy:

a) *Electrical implants such as cardiac pacemakers.*
b) *Ferromagnetic implants such as aneurysm clips, surgical clips, artificial hearts valves with steel parts, shrapnel or steel implants.*
c) *Ferromagnetic objects such as jewelry or metal clips in clothing.*
d) *Pre-existing history of claustrophobic reactions.*

Adequacy of Protection against Risk

Recruitment and Informed Consent: Potential patients will be identified from oncology clinics, urology clinics and the community. An IRB approved letter and flyer will also be sent to all full-time and part-time Brigham and Women's Hospital and Dana Farber Cancer Institute's oncology and urology faculty members and to community oncologists and urologists. Furthermore, the study will be advertised in various media outlets such as newspapers, magazines, internet sources (i.e. https://clinicaltrials.partners.org/), and radio. If a subject qualifies for the study based on personal interviews or telephone contact, informed consent will be obtained by the study coordinator and/or principal investigator. All the subjects will be explained in detail the research protocol, benefits and risks. The subjects will be explained that the participation in this study is voluntary and that they may withdraw at any time. All the questions by the participants will be answered. All the procedures will be HIPAA compliant as outlined in Brigham and Women's Hospital policy procedures.

Protection against Risk: There are only minimal risks associated with the study. Trained technicians will perform phlebotomies and experienced investigators will supervise the oral glucose tolerance test and the MRS procedures.

Potential Benefits to the Subjects

Each subject will receive a total of $350.00 (dispensed at various visits) for their time and effort in accordance with the Partners IRB guidelines. All participants will also receive parking vouchers and meal coupons to have breakfast after fasting blood draws.

During this study, participants will receive a free evaluation of many aspects of their health. Since patients are not blinded, they will be provided information regarding the results in real time. Patients will get reports regarding their anthropometry, vital signs, plasma glucose, lipids and other chemistries at each visit. One of the main benefits that the patients may obtain will be from knowing if they have underlying diabetes at baseline. They may also derive satisfaction from the knowledge that their participation has contributed to the greater understanding of the link between insulin resistance and ADT. This information will even help them to discuss these issues with their oncologists and make informed decisions regarding their therapy.

REFERENCES

1. Siegel RL, Miller KD, Jemal A. Cancer statistics, 2017. *CA Cancer J Clin.*2017;67:7-30.
2. Huggins C, Stevens RE Jr, Hodges CV. Studies on prostatic cancer. II. The effects of castration on advanced carcinoma of the prostate gland. *Arch Surg.*1941;43:209-23.
3. Smith MR. Androgen deprivation therapy for prostate cancer: new concepts and concerns. *Curr Opin Endocrinol Diabetes Obes*. 2007;14:247–254.
4. Basaria S, Bhasin S. Cardiometabolic complications of androgen deprivation therapy in prostate cancer: Targeting the Skeletal Muscle-Metabolism Axis. *N Engl J Med*. 2012;367:965-7.
5. Basaria S. Time to raise awareness regarding complications of androgen deprivation therapy. *Asian J Androl*. 2012;14:175-76.
6. Harle LK, Maggio M, Shahani S, Braga-Basaria M, Basaria S. Endocrine complications of androgen-deprivation therapy in men with prostate cancer. *Clin Adv Hematol Oncol*. 2006;4:687-96.
7. Basaria S, Lieb 2nd J, Tang A, et al. Long-term effects of androgen deprivation therapy in prostate cancer patients. *Clin Endocrinol (Oxf)*. 2002;56:779–786.
8. Hamilton EJ, Gianatti E, Strauss BJ, Wentworth J, Lim-Joon D, Bolton D, Zajac JD, Grossmann M. Increase in visceral and subcutaneous abdominal fat in men with prostate cancer treated with androgen deprivation therapy.*Clin Endocrinol (Oxf)*. 2011;74:377-83.
9. Bressler P, Bailey SR, Matsuda M, DeFronzo RA. Insulin resistance and coronary artery disease.*Diabetologia*. 1996;39:1345-50.
10. Haffner SM. Epidemiology of insulin resistance and its relation to coronary artery disease.*Am J Cardiol*. 1999;84:11J-14J.
11. Hanley AJ, Williams K, Stern MP, Haffner SM. Homeostasis model assessment of insulin resistance in relation to the incidence of cardiovascular disease: the San Antonio Heart Study.*Diabetes Care*. 2002;25:1177-84.
12. Kraemer FB, Ginsberg HN. Demonstration of the Central Role of Insulin Resistance in Type 2 Diabetes and Cardiovascular Disease.*Diabetes Care*. 2014;37:1178-81.
13. Salazar MR, Carbajal HA, Espeche WG, Dulbecco CA, Aizpurúa M, Marillet AG, Echeverría RF, Reaven GM. Relationships among insulin resistance, obesity, diagnosis of the metabolic syndrome and cardio-metabolic risk.*Diab Vasc Dis Res*. 2011;8:109-16.
14. Reaven GM. Insulin resistance: the link between obesity and cardiovascular disease.*Med Clin North Am*. 2011;95:875-92.
15. Defronzo RA, Tripathy D, Schwenke DC, Banerji M, Bray GA, Buchanan TA, Clement SC, Henry RR, Kitabchi AE, Mudaliar S, Ratner RE, Stentz FB, Musi N, Reaven PD, Gastaldelli A. Prediction of diabetes based on baseline metabolic characteristics in individuals at high risk.*Diabetes Care*. 2013;36:3607-12.
16. Basaria S, Muller DC, Carducci MA, et al. Hyperglycemia and insulin resistance in men with prostate carcinoma who receive androgen deprivation therapy. *Cancer.* 2006; 106:581-588.
17. Braga-Basaria M, Dobs AS, Muller DC, Carducci MA, John M, Egan J, Basaria S. Metabolic syndrome in men with prostate cancer undergoing long-term androgen-deprivation therapy. *J Clin Oncol.* 2006; 24:3979-3983.
18. Basaria S, Muller DC, Carducci MA, Egan J, Dobs AS. Relation between duration of androgen deprivation therapy and degree of insulin resistance in men with prostate cancer. *Arch Intern Med.*2007;167:612-3.

19. Shahani S, Braga-Basaria M, Basaria S. Androgen deprivation therapy in prostate cancer and metabolic risk for atherosclerosis. *J Clin Endocrinol Metab.* 2008;93:2042-9.
20. Basaria S, Dobs AS. Testosterone making an entry into the cardiometabolic world. *Circulation*. 2007;116:2658-61.
21. Smith JC, Bennett S, Evans LM, et al. The effects of induced hypogonadism on arterial stiffness, body composition, and metabolic parameters in males with prostate cancer. *J Clin Endocrinol Metab.* 2001;86:4261-4267.
22. Dockery F, Bulpitt CJ, Agarwal S, et al. Testosterone suppression in men with prostate cancer leads to an increase in arterial stiffness and hyperinsulinaemia. *Clin Sci (Lond).* 2003; 104:195-201.
23. Smith MR, Lee H, Nathan DM. Insulin sensitivity during combined androgen blockade for prostate cancer. *J Clin Endocrinol Metab.* 2006; 91:1305-1308.
24. Keating NL, O'Malley AJ, Smith MR. Diabetes and cardiovascular disease during androgen deprivation therapy for prostate cancer. *J Clin Oncol*. 2006;24: 4448-4456.
25. Hu JC, Williams SB, O'Malley AJ, Smith MR, Nguyen PL, Keating NL. Androgen-deprivation therapy for nonmetastatic prostate cancer is associated with an increased risk of peripheral arterial disease and venous thromboembolism.*Eur Urol*. 2012;61:1119-28.
26. Satariano WA, Ragland KE, van den Eeden SK. Cause of death in men diagnosed with prostate carcinoma. *Cancer*. 1998;83:1180-8.
27. Lu-Yao G, Stukel TA, Yao SL. Changing patterns in competing causes of death in men with prostate cancer: a population based study. *J Urol*2004;171:2285-90.
28. Henry RR. Type 2 diabetes care: the role of insulin-sensitizing agents and practical implications for cardiovascular disease prevention.*Am J Med.* 1998;105:20S-26S.
29. Wang H, Knaub LA, Jensen DR, et al. Skeletal muscle-specific deletion of lipoprotein lipase enhances insulin signaling in skeletal muscle but causes insulin resistance in liver and other tissues.*Diabetes*. 2009;58:116-24.
30. Kotronen A, Seppälä-Lindroos A, Bergholm R, Yki-Järvinen H. Tissue specificity of insulin resistance in humans: fat in the liver rather than muscle is associated with features of the metabolic syndrome.*Diabetologia*. 2008;51:130-8.
31. Seppälä-Lindroos A, Vehkavaara S, Häkkinen AM, et al. Fat accumulation in the liver is associated with defects in insulin suppression of glucose production and serum free fatty acids independent of obesity in normal men.*J Clin Endocrinol Metab*. 2002;87:3023-8.
32. Petersen KF, Shulman GI. Etiology of insulin resistance.*Am J Med*. 2006;119:S10-6.
33. Abdul-Ghani MA, Matsuda M, Balas B, DeFronzo RA. Muscle and liver insulin resistance indexes derived from the oral glucose tolerance test.*Diabetes Care*. 2007;30:89-94.
34. Hotamisligil GS. Inflammation and metabolic disorders. Nature. 2006;444:860-7.
35. Ingram KH, Hill H, Moellering DR, et al. Skeletal muscle lipid peroxidation andinsulin resistance in humans.*J Clin Endocrinol Metab*. 2012 ;97:E1182-6.
36. Hotamisligil GS, Murray DL, Choy LN, Spiegelman BM. Tumor necrosis factor alpha inhibits signaling from the insulin receptor.*Proc Natl Acad Sci* U S A. 1994;91:4854-8.
37. Muoio DM. Revisiting the connection between intramyocellular lipids andinsulin resistance: a long and winding road.*Diabetologia*. 2012;55:2551-4.
38. Hotamisligil GS, Arner P, Caro JF, Atkinson RL, Spiegelman BM. Increased adipose tissue expression of tumor necrosis factor-alpha in human obesity and insulin resistance.*J Clin Invest*. 1995;95:2409-15.
39. Kotronen A, Juurinen L, Tiikkainen M, Vehkavaara S, Yki-Järvinen H. Increased liver fat, impaired insulin clearance, and hepatic and adipose tissue insulin resistance in type 2 diabetes.*Gastroenterology*. 2008;135:122-30.

40. Hotamisligil GS, Shargill NS, Spiegelman BM. Adipose expression of tumor necrosis factor-alpha: direct role in obesity-linked insulin resistance.*Science*.1993;259(5091):87-91.
41. Lettner A, Roden M. Ectopic fat and insulin resistance.*Curr Diab Rep*. 2008;8:185-91.
42. Solomon DH, Massarotti E, Garg R, Liu J, Canning C, Schneeweiss S.Association between disease-modifying antirheumatic drugs and diabetes risk in patients with rheumatoid arthritis and psoriasis.*JAMA*. 2011;305:2525-31.
43. Stanley TL, Zanni MV, Johnsen S, et al. TNF-alpha antagonism with etanercept decreases glucose and increases the proportion of high molecular weight adiponectin in obese subjects with features of the metabolic syndrome.*J Clin Endocrinol Metab*. 2011;96:E146-50.
44. Bernstein LE, Berry J, Kim S, Canavan B, Grinspoon SK. Effects of etanercept in patients with the metabolic syndrome.*Arch Intern Med*. 2006;166:902-8.
45. Bylow K, Dale W, Mustian K, et al. Falls and physical performance deficits in older patients with prostate cancer undergoing androgen deprivation therapy.*Urology*. 2008;72:422-7.
46. Bylow K, Hemmerich J, Mohile SG, et al. Obese frailty, physical performance deficits, and falls in older men with biochemical recurrence of prostate cancer on androgen deprivation therapy: a case-control study.*Urology*. 2011;77:934-40.
47. Basaria S, Collins L, Dillon EL, et al. The safety, pharmacokinetics, and effects of LGD-4033, a novel nonsteroidal oral, selective androgen receptor modulator, in healthyyoung men.*J Gerontol A Biol Sci Med Sci*. 2013;68:87-95.
48. Huggins C, Hodges CV. Studies on prostatic cancer. The effect of castration, of estrogen and androgen injection on serum phosphatases in metastatic carcinoma of the prostate.*CA Cancer J Clin*. 1972;22:232-40.
49. Bubley GJ, Carducci M, Dahut W et al. Eligibility and response guidelines for phase II clinical trials in androgen-independent prostate cancer: recommendations from the Prostate-Specific Antigen Working Group. *J Clin Oncol* 1999; 17:3461-3467.
50. Connolly RM, Carducci MA, Antonarakis ES. Use of androgen deprivation therapy in prostate cancer: indications and prevalence.*Asian J Androl*. 2012;14:177-86.
51. Sharifi N, Gulley JL, Dahut WL.Androgen deprivation therapy for prostate cancer.*JAMA*. 2005;294:238-44.
52. Collins L,Basaria S. *Asian J Androl*. 2012;14:222-5.
53. Collins L, Mohammed N, Ahmad T, Basaria S. Androgen deprivation therapy for prostate cancer: implications for cardiometabolic clinical care.*J Endocrinol Invest*. 2012;35:332-9.
54. Shahinian VB, Kuo YF, Freeman JL, Orihuela E, Goodwin JS. Increasing use of gonadotropin-releasing hormone agonists for the treatment of localized prostate carcinoma.*Cancer*. 2005;103:1615-24.
55. Levine GN, D'Amico AV, Berger P, et al. Androgen-deprivation therapy in prostate cancer and cardiovascular risk: a science advisory from the American Heart Association, American Cancer Society, and American Urological Association: endorsed by the American Society for Radiation Oncology. *Circulation.* 2010;121:833-40.
56. Azoulay L, Yin H, Benayoun S, Renoux C, Boivin JF, Suissa S. Androgen deprivation therapy and the risk of stroke in patients with prostate cancer. *Euro Urol*. 2011;60: 1244-50.
57. Basaria S. Prostate cancer: Cardiovascular mortality and androgen deprivation. *Nat Rev Urol.*2009;6:252-3.

58. Bhasin S, Taylor WE, Singh R, et al. The mechanisms of androgen effects on body composition: mesenchymal pluripotent cell as the target of androgen action. *J Gerontol A Biol Sci Med Sci*. 2003;58:M1103-10.
59. Singh R, Artaza JN, Taylor WE, Gonzalez-Cadavid NF, Bhasin S. Androgens stimulate myogenic differentiation and inhibit adipogenesis in C3H 10T1/2 pluripotent cells through an androgen receptor-mediated pathway. *Endocrinology*. 2003;144:5081-8.
60. Singh R, Bhasin S, Braga M, et al. Regulation of myogenic differentiation by androgens: cross talk between androgen receptor/ beta-catenin and follistatin/transforming growth factor-beta signaling pathways. *Endocrinology*. 2009;150:1259-68.
61. Basaria S, Dobs AS. Hypogonadism and androgen replacement therapy in elderly men. *Am J Med.* 2001;110:563-72.
62. Basaria S. Male hypogonadism. *Lancet*. 2014;383:1250-63.
63. Mauras N, Hayes V, Welch S, et al. Testosterone deficiency in young men: marked alterations in whole body protein kinetics, strength, and adiposity. *J Clin Endocrinol Metab.* 1998;83:1886-92.
64. Bhasin S, Basaria S. Diagnosis and treatment of hypogonadism in men. *Best Pract Res Clin Endocrinol Metab*. 2011;25:251-70.
65. Katznelson L, Finkelstein JS, Schoenfeld DA, et al. Increase in bone density and lean body mass during testosterone administration in men with acquired hypogonadism. *J Clin Endocrinol Metab.* 1996;81:4358-65.
66. Brodsky IG, Balagopal P, Nair KS. Effects of testosterone replacement on muscle mass and muscle protein synthesis in hypogonadal men--a clinical research center study. *J Clin Endocrinol Metab.* 1996;81:3469-75.
67. Bhasin S, Woodhouse L, Casaburi R, et al. Older men are as responsive as young men to the anabolic effects of graded doses of testosterone on the skeletal muscle. *J Clin Endocrinol Metab.* 2005;90:678-88.
68. Bhasin S, Travison TG, Storer TW, Lakshman K, Kaushik M, Mazer NA, Ngyuen AH, Davda MN, Jara H, Aakil A, Anderson S, Knapp PE, Hanka S, Mohammed N, Daou P, Miciek R, Ulloor J, Zhang A, Brooks B, Orwoll K, Hede-Brierley L, Eder R, Elmi A, Bhasin G, Collins L, Singh R, Basaria S. Effect of testosterone supplementation with and without a dual 5α-reductase inhibitor on fat-free mass in men with suppressed testosterone production: a randomized controlled trial. *JAMA*. 2012;307:931-9.
69. Wajchenberg BL. Subcutaneous and visceral adipose tissue: their relation to the metabolic syndrome. *Endocr Rev*. 2000;21:697-738.
70. Smith MR, Finkelstein JS, McGovern FJ, et al. Changes in body composition during androgen deprivation therapy for prostate cancer. *J Clin Endocrinol Metab.* 2002; 87:599-603.
71. Chen Z, Maricic M, Nguyen P, Ahmann FR, Bruhn R, Dalkin B. Low bone density and high percentage of body fat among men who were treated withandrogen deprivation therapy for prostate carcinoma. *Cancer.* 2002; 95:2136-2144.
72. Muller M, Grobbee DE, den Tonkelaar I, et al. Endogenous sex hormones and metabolic syndrome in aging men. *J Clin Endocrinol Metab.* 2005; 90:2618-2623.
73. Haffner SM, Shaten J, Stern MP, et al. Low levels of sex hormone-binding globulin and testosterone predict the development of non-insulin-dependent diabetes mellitus in men. MRFIT Research Group. Multiple Risk Factor Intervention Trial. *Am J Epidemiol.*1996;143:889- 897.

74. Selvin E, Manning F, Zhang L, Rohrmann S, Rifai N, Nelson WG, Dobs A, Basaria S, Golden SH, Platz EA. Androgens and diabetes in men: Results from the third National Health and Nutrition Examination Survey (NHANES III). *Diabetes Care*2007;30:234-38.
75. Stellato RK, Feldman HA, Hamdy O, Horton ES, McKinlay JB. Testosterone, sex hormone-binding globulin, and the development of type 2 diabetes in middle-aged men: prospective results from the Massachusetts male aging study.*Diabetes Care*. 2000;23:490-4.
76. Kupelian V, Hayes FJ, Link CL, Rosen R, McKinlay JB. Inverse association of testosterone and the metabolic syndrome in men is consistent across race and ethnic groups.*J Clin Endocrinol Metab*. 2008;93:3403-10.
77. Kupelian V, Page ST, Araujo AB, Travison TG, Bremner WJ, McKinlay JB. Low sex hormone-binding globulin, total testosterone, and symptomatic androgen deficiency are associated with development of the metabolic syndrome in nonobese men.*J Clin Endocrinol Metab*. 2006;91:843-50.
78. Maggio M, Basaria S. Welcoming low testosterone as a cardiovascular risk factor. *Int J Impot Res*. 2009;21:261-4.
79. Laaksonen DE, Niskanen L, Punnonen K, et al. Testosterone and sex hormone-binding globulin predict the metabolic syndrome and diabetes in middle-aged men. *Diabetes Care.* 2004; 27:1036-1041.
80. Derweesh IH, Diblasio CJ, Kincade MC, et al. Risk of new-onset diabetes mellitus and worsening glycaemic variables for established diabetes in men undergoing androgen-deprivation therapy for prostate cancer.*BJU Int*. 2007;100:1060-5.
81. Ford ES, Giles WH, Dietz WH. Prevalence of the metabolic syndrome among US adults: findings from the third National Health and Nutrition Examination Survey. *JAMA*. 2002;287:356-9.
82. Reaven GM. Role of insulin resistance in human disease. *Diabetes*. 1988;37:1595-607.
83. Saigal CS, Gore JL, Krupski TL, et al. Androgen deprivation therapy increases cardiovascular morbidity in men with prostate cancer. *Cancer.* 2007;110:1493-500.
84. D'Amico AV, Denham JW, Crook J, et al. Influence of androgen suppression therapy for prostate cancer on the frequency and timing of fatal myocardial infarctions. *J Clin Oncol*. 2007;25:2420-5.
85. Tsai HK, D'Amico AV, Sadetsky N, Chen MH, Carroll PR. Androgen deprivation therapy for localized prostate cancer and the risk of cardiovascular mortality. *J Natl Cancer Inst*. 2007;99:1516-24.
86. Nanda A, Chen MH, Braccioforte MH, Moran BJ, D’Amico AV. Hormonal therapy use for prostate cancer and mortality in men with coronary artery disease-induced congestive heart failure or myocardial infarction. *JAMA*. 2009;302:866-73.
87. Lazar MA. Resistin- and obesity-associated metabolic diseases. *Horm Metab Res*. 2007;710-6.
88. Reilly MP, Lehrke M, Wolfe ML, Rohatgi A, Lazar MA, Rader DJ. Resistin is an inflammatory marker of atherosclerosis in humans.*Circulation*. 2005;111:932-9.
89. Glass CK, Olefsky JM. Inflammation and lipid signaling in the etiology of insulin resistance.*Cell Metab*. 2012;15:635-45.
90. Jornayvaz FR, Shulman GI. Diacylglycerol activation of protein kinase Cε and hepatic insulin resistance.*Cell Metab*. 2012;15:574-84.
91. Samuel VT, Shulman GI. Mechanisms for insulin resistance: common threads and missing links.*Cell*. 2012;148:852-71.
92. Shulman GI.Cellular mechanisms of insulin resistance.*J Clin Invest*. 2000 ;106:171-6.

93. Johnson AM, Olefsky JM. The origins and drivers of insulin resistance.*Cell*. 2013;152:673-84.
94. Olefsky JM. Fat talks, liver and muscle listen. *Cell*. 2008;134:914-6.
95. Bizzarro A, Valentini G, Di Martino G, DaPonte A, De Bellis A, Iacono G. Influence of testosterone therapy on clinical and immunological features of autoimmune diseases associated with Klinefelter's syndrome. *J Clin Endocrinol Metab*. 1987;64:32-6.
96. Spinedi E, Suescun MO, Hadid R, Daneva T, Gaillard RC. Effects of gonadectomy and sex hormone therapy on the endotoxin-stimulated hypothalamo-pituitary-adrenal axis: evidence for a neuroendocrine-immunological sexual dimorphism. *Endocrinology*. 1992;131:2430-6.
97. Maggio M, Basaria S, Ble A, Lauretani F, Bandinelli S, Ceda GP, et al. Correlation between testosterone and the inflammatory marker soluble interleukin-6 receptor in older men. *J Clin Endocrinol Metab*. 2006;91:345-7.
98. Khosla S, Atkinson EJ, Dunstan CR, O'Fallon WM. Effect of estrogen versus testosterone on circulating osteoprotegerin and other cytokine levels in normal elderly men. *J Clin Endocrinol Metab*. 2002;87:1550-4.
99. Malkin CJ, Pugh PJ, Jones RD, Kapoor D, Channer KS, Jones TH. The effect of testosterone replacement on endogenous inflammatory cytokines and lipid profiles in hypogonadal men. *J Clin Endocrinol Metab*. 2004;89:3313-8.
100. Garg R, Tripathy D, Dandona P. Insulin resistance as a proinflammatory state: mechanisms, mediators, and therapeutic interventions.*Curr Drug Targets*. 2003;4:487-92.
101. Pradhan AD, Manson JE, Rifai N, Buring JE, Ridker PM. C-reactive protein, interleukin 6, and risk of developing type 2 diabetes mellitus.*JAMA*. 2001;286:327-34.
102. Osborn O, Olefsky JM. The cellular and signaling networks linking the immune system and metabolism in disease.*Nat Med*. 2012;18:363-74.
103. Gonzalez-Gay MA, De Matias JM, Gonzalez-Juanatey C, et al. Anti-tumor necrosis factor-alpha blockade improves insulin resistance in patients with rheumatoid arthritis.*Clin Exp Rheumatol*. 2006;24:83-6.
104. Holmäng A, Björntorp P. The effects of testosterone on insulin sensitivity in male rats. *Acta Physiol Scand.*1992;146:505-10.
105. Sinha-Hikim I, Artaza J, Woodhouse L, et al. Testosterone-induced increase in muscle size in healthy young men is associated with muscle fiber hypertrophy. *Am J Physiol Endocrinol Metab.* 2002;283:E154-64.
106. Lillioja S, Young AA, Culter CL, et al. Skeletal muscle capillary density and fiber type are possible determinants of in vivo insulin resistance in man. *J Clin Invest.* 1987;80:415-24.
107. Mårin P, Högh-Kristiansen I, Jansson S, et al. Uptake of glucose carbon in muscle glycogen and adipose tissue triglycerides in vivo in humans. *Am J Physiol.* 1992;263:E473-80.
108. Petersen KF, Dufour S, Savage DB, et al. The role of skeletal muscle insulin resistance in the pathogenesis of the metabolic syndrome.*Proc Natl Acad Sci*. 2007;104:12587-94.
109. Abdul-Ghani MA, DeFronzo RA. Pathogenesis of insulin resistance in skeletal muscle.*J Biomed Biotechnol*. 2010;:476279.
110. Matsuda M, DeFronzo RA. Insulin sensitivity indices obtained from oral glucose tolerance testing: comparison with the euglycemic insulin clamp.*Diabetes Care*. 1999;22:1462-70.

111. Stumvoll M, Mitrakou A, Pimenta W, et al. Use of the oral glucose tolerance test to assess insulin release and insulin sensitivity. *Diabetes Care*. 2000;23:295-301.

112. American Diabetes Association. Diagnosis and classification of diabetes mellitus. *DiabetesCare*. 2014;37:S81-90.

113. Matthews DR, Hosker JP, Rudenski AS, et al. Homeostasis model assessment: insulin resistance and beta-cell function from fasting plasma glucose and insulin concentrations in man. *Diabetologia*. 1985;28:412-9.

114. Petersen KF, Shulman GI. New insights into the pathogenesis of insulin resistance in humans using magnetic resonance spectroscopy. *Obesity (Silver Spring).* 2006;14:34S-40S.

115. Shulman GI. Unraveling the cellular mechanism of insulin resistance in humans: new insights from magnetic resonance spectroscopy. *Physiology (Bethesda)*. 2004;19:183-90.

116. Knowler WC, Barrett-Connor E, Fowler SE, et al. Reduction in the incidence of type 2 diabetes with lifestyle intervention or metformin. *N Engl J Med*. 2002;346:393-403.

117. Buchanan TA, Xiang AH, Peters RK, et al. Preservation of pancreatic beta-cell function and prevention of type 2 diabetes by pharmacological treatment of insulin resistance in high-risk hispanic women. *Diabetes*. 2002;51:2796-803.

118. Dormandy JA, Charbonnel B, Eckland DJ, et al. Secondary prevention of macrovascular events in patients with type 2 diabetes in the PROactive Study (PROspective pioglitAzone Clinical Trial In macroVascular Events): a randomized controlled trial. *Lancet*. 2005;366:1279-89.

119. UKPDS-34. Effect of intensive blood-glucose control with metformin on complications in overweight patients with type 2 diabetes (UKPDS 34). UK Prospective Diabetes Study (UKPDS) Group. *Lancet*. 1998;352:854-65.

120. Stumvoll M, Meyer C, Mitrakou A, Nadkarni V, Gerich JE. Renal glucose production and utilization: new aspects in humans. *Diabetologia*. 1997;40:749-57.

121. DeFronzo RA, Ferrannini E, Hendler R, Felig P, Wahren J. Regulation of splanchnic and peripheral glucose uptake by insulin and hyperglycemia in man. Diabetes. 1983 Jan;32(1):35-45.

122. Ferrannini E, Bjorkman O, Reichard GA Jr, et al. The disposal of an oral glucose load in healthy subjects. A quantitative study. *Diabetes*. 1985;34:580-8.

123. Jara H, Soto JA, Yu B, et al. Multisection T1-weighted hybrid-RARE: a pulse sequence for MR imaging of the entire liver during suspended respiration. *Magn Reson Med*. 1996;36:767-74.

124. Lee VS, Lavelle MT, Krinsky GA, Rofsky NM. Volumetric MR imaging of the liver and applications. *Magn Reson Imaging Clin N Am*. 2001;9:697-716.

125. Ortiz-Lopez C, Lomonaco R, Orsak B, et al. Prevalence of prediabetes and diabetes and metabolic profile of patients with nonalcoholic fatty liver disease (NAFLD). *Diabetes Care.* 2012;35:873-8.

126. Belfort R, Harrison SA, Brown K, et al. A placebo-controlled trial of pioglitazone in subjects with nonalcoholic steatohepatitis. *N Eng J Med* 2006;355:2297-307.

127. Szczepaniak LS, Nurenberg P, Leonard D, et al. Magnetic resonance spectroscopy to measure hepatic triglyceride content: prevalence of hepatic steatosis in the general population. Am J Physiol Endocrinol Metab. 2005;288:E462-8.

128. Johnson NA, Walton DW, Sachinwalla T, et al. Noninvasive assessment of hepatic lipid composition: Advancing understanding and management of fatty liver disorders. *Hepatology* 2008;47:1513-23.

129. van Werven JR, Schreuder TC, Nederveen AJ, et al. Hepatic unsaturated fatty acids in patients with non-alcoholic fatty liver disease assessed by 3.0T MR spectroscopy. *Eur J Radiol*. 2010;75:102-7.

130. Cobbold JF, Anstee QM, Goldin RD, et al. Phenotyping murine models of non-alcoholic fatty liver disease through metabolic profiling of intact liver tissue. *Clin Sci (Lond)*. 2009;116:403-13.

131. Boesch C. Musculoskeletal spectroscopy. *J Magn Reson Imaging.* 2007;25:321-38.

132. Spitzer M, Bhasin S, Travison TG, Davda MN, Stroh H, Basaria S. Sildenafil increases serum testosterone levels by a direct action on the testes.*Andrology*. 2013;1:913-8.

133. Spitzer M, Basaria S, Travison TG, et al. Effect of testosterone replacement on response to sildenafil citrate in men with erectile dysfunction: a parallel, randomized trial.*Ann Intern Med*. 2012;157:681-91.

134. Huang G, Tang E, Aakil A, Anderson S, Jara H, Davda M, Stroh H, Travison TG, Bhasin S, Basaria S. Testosterone Dose-Response Relationships with Cardiovascular Risk Markers in Androgen-Deficient Women: A Randomized, Placebo-Controlled Trial.*J Clin Endocrinol Metab*. 2014; E1287-93.

135. Topolski TD, LoGerfo J, Patrick DL, Williams B, Walwick J, Patrick MB. The rapid assessment of physical activity (RAPA) among older adults. *Prev Chronic Dis*. 2006; 3:1-8.

136. Bhasin S, Apovian CM, Travison TG, Pencina K, Huang G, Moore LL, Campbell WW, Howland A, Chen R, Singer MR, Shah M, Eder R, Schram H, Bearup R, Beleva YM, McCarthy AC, Li Z, Woodbury E, McKinnon J, Storer TW, Basaria S.Design of a randomized trial to determine the optimum protein intake to preserve lean body mass and to optimize response to a promyogenic anabolic agent in older men with physical function limitation. *Contemp Clin Trials*. 2017;58:86-93.

137. Willett WC, Sampson L, Stampfer MJ, Rosner B, Bain C, Witschi J, Hennekens CH, Speizer FE. Reproducibility and validity of a semiquantitative food frequency questionnaire. *Am J Epidemiol*. 1985; 122:51-65.

138. Rimm EB, Giovannucci EL, Stampfer MJ, Colditz GA, Litin LB, Willett WC. Reproducibility and validity of a semiquantitative food frequency questionnaire among male health professionals. *Am J Epidemiol*. 1992; 135:1114-26.

139. Newby PK, Hu FB, Rimm EB, Smith-Warner SA, Feskanich D, Sampson L, Willett WC. Reproducibility and validity of the Diet Quality Index as assessed by use of a food frequency questionnaire. *Am J Clin Nutr*. 2003; 78:941-49.

140. Glymour MM. When Is Baseline Adjustment Useful in Analyses of Change? An Example with Education and Cognitive Change. *American Journal of Epidemiology* 2005;162, 267–278.

141. Fitzmaurice GM, Laird NM, Ware JH. *Applied Longitudinal Analysis* (Wiley-Interscience, 2004).

142. Rothman K. J. Six Persistent Research Misconceptions. *Journal of General Internal Medicine.* 2014;11606-013-2755.

143. Wood S. *Generalized Additive Models: An Introduction with R*. (Chapman and Hall/CRC, 2006).

144. Zou GY, Donner A. Extension of the modified Poisson regression model to prospective studies with correlated binary data. *Stat Methods Med Res.* 2013;22:661-70.

145. Little RJ, Wang Y. Pattern-Mixture Models for Multivariate Incomplete Data with Covariates. *Biometrics.* 1996;52:98.

146. Hayes RP, Ni X, Heiselman DE, Kinchen K. Psychometric testing of two new patient-reported outcome instruments for the evaluation of treatment for hypogonadism. Int J Clin Pract. 2016; 70:587-95.

147. Hayes RP, Henne J, Kinchen K. Establishing the content validity of the Sexual Arousal, Interest and Drive Scale and the Hypogonadism Energy Diary. Int J Clin Pract. 2015; 69:454-65.
